# Scalable manufacture of nearly pure-phase metallic MoS_2_ nanosheets

**DOI:** 10.1038/s41563-026-02480-2

**Published:** 2026-01-29

**Authors:** Ziwei Jeffrey Yang, Zhuangnan Li, Leyi Loh, James Moloney, John Walmsley, Jiahang Li, Yuan Chen, Lixin Liu, Han Zang, Han Yan, Soumya Sarkar, Jason Day, Yan Wang, Manish Chhowalla

**Affiliations:** 1 Department of Materials Science & Metallurgy, University of Cambridge, Cambridge, UK; 2 College of Chemical and Biological Engineering, Zhejiang University, Hangzhou, China; 3 Department of Physics, National University of Singapore, Singapore, Singapore; 4 Department of Earth Sciences, University of Cambridge, Cambridge, UK

**Keywords:** Nanoscale materials, Materials for energy and catalysis

## Abstract

Metallic, two-dimensional molybdenum disulfide (MoS_2_) nanosheets show promise for energy storage and catalysis applications. However, current chemical exfoliation methods require more than 48 h to produce milligrams of material, and result in an impure mixture of metallic (1T/1T′, approximately 50%–70%) and semiconducting (2H) phases. Here we demonstrate large-scale and rapid (>600 g h^−1^) production of nearly pure-phase metallic two-dimensional MoS_2_ nanosheets using microwave irradiation. Atomic-resolution imaging and X-ray photoelectron spectroscopy show nearly 100% metallic phase in the basal plane. This high purity leads to a large exchange current density (0.175 ± 0.030 mA cm^−2^) and low Tafel slopes (39–47 mV dec^−1^) for hydrogen evolution reaction. In supercapacitors and lithium–sulfur pouch-cell batteries, the resulting nanosheets enable a high volumetric capacitance of 753.0 ± 3.6 F cm^−3^ and a specific capacity of 1,245 ± 16 mAh g^−1^ (electrolyte-to-sulfur ratio, 2 µl mg^−1^), respectively. Our method provides a practical pathway for producing high-quality metallic two-dimensional materials for high-performance energy devices.

## Abstract (web summary)

A microwave-assisted process is developed for the rapid and scalable manufacture of pure-phase metallic MoS_2_ nanosheets, enabling practical electrochemical devices for energy applications.

## Main

The metallic 1T(1T′) phase of molybdenum disulfide (MoS_2_) is interesting for applications in catalysis^1–4^ and energy storage^5,6^. However, to translate the excellent performance from proof-of-concept devices with small-scale (milligram (mg)) samples into practical demonstrations, large-scale (kilogram (kg)) and rapid production of high-quality materials is required. Bulk crystals of metallic transition metal dichalcogenides (TMDs) have been recently synthesized^7,8^. Although metallic layered crystals can be exfoliated into atomically thin nanosheets^9^, producing them at scale sufficient for electrochemical applications and retaining a high metallic phase concentration remains a challenge^10^.

Chemical exfoliation of TMDs with *n*-butyllithium (*n*-BuLi) in hexane has been demonstrated for the synthesis of metallic nanosheets^11,12^. The yield of single-layer nanosheets is nearly 100% with this method. However, two challenges have hampered the use of this method for the large-scale production of metallic nanosheets. The first is that the as-exfoliated nanosheets are a mixture of metallic 1T(1T′) and semiconducting 2H phases—with the 1T(1T′)-phase concentration being 50%–70% for typical reaction conditions^3,12–14^. The second is that the reaction is slow, requiring 48–72 h for the homogeneous synthesis of tens of milligrams of nanosheets^11,12,15–17^. These issues have limited their implementation into practical energy devices in which large quantities (kilogram to ton scale) are required^18^.

Efforts to accelerate the chemical exfoliation process lead to the deterioration of nanosheet properties. For example, increasing the *n*-BuLi concentration leads to overlithiation, resulting in the decomposition of MoS_2_ into Mo metal and lithium sulfide (Li_2_S)^19,20^. Increasing the reaction temperature can enhance the reaction kinetics. However, due to the strong reducing and deprotonating effects of *n*-BuLi, synthesis is typically performed in hexane for chemical stability. This restricts the reaction temperature to the boiling point of hexane (66 °C), thereby limiting the reaction and 1T(1T′)-phase conversion rates. One reported modification to the chemical exfoliation (CE) process enables heating to 100 °C, thereby reducing the reaction time. However, this requires a pressurized autoclave reactor and careful pressure moderation^9^.

**Fig. 1:**
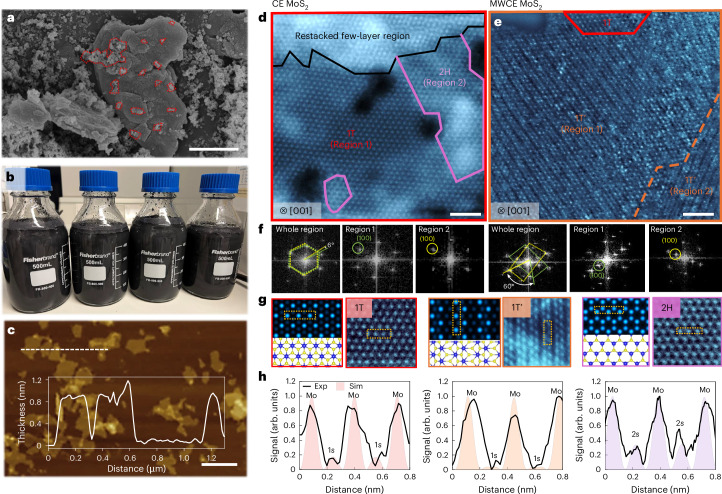
Imaging of CE and MWCE MoS_2_. **a**, Scanning electron microscopy image of the precursor MoS_2_ with ~100-nm carbon black susceptor nanoparticles (highlighted with the red dotted lines). Scale bar, 1 µm. **b**, Photograph of 100 g of MWCE MoS_2_ nanosheets dispersed in deionized water. **c**, AFM image of individual exfoliated MoS_2_ nanosheets, and height profile along the dotted white line. Scale bar, 500 nm. **d**, Typical ADF-STEM image of CE MoS_2_, showing mixed 2H, 1T and 1T′ phases. Scale bar, 2 nm. **e**, ADF-STEM image of MWCE MoS_2_, showing only 1T/1T′ phases. Scale bar, 2 nm. **f**, Fast Fourier transform of the entire region and selected regions of the STEM images shown above. **g**, Enlarged 1T, 1T′ and 2H phases with their corresponding simulated images overlaid with the atomic models. **h**, *Z*-contrast intensity profiles along the atoms in the yellow rectangles of the 1T, 1T′ and 2H phases, from both simulated and experimental images. Sulfur signals in the 1T/1T′ phases are much lower relative to those in the 2H phase.

In this work, we use localized microwave heating for the fast synthesis of high-purity metallic 1T(1T′)-phase TMDs such as MoS_2_, tungsten disulfide (WS_2_) and molybdenum diselenide (MoSe_2_). Previous reports on the microwave chemical exfoliation (MWCE) of MoS_2_ yielded a relatively low concentration of the metallic 1T(1T′) phase even at long irradiation times^21^. This is because the starting 2H-phase MoS_2_ lacks defects, functional groups and electrical conductivity to effectively induce dipole polarization and Joule heating under microwave irradiation^22^. Here we use carbon black nanoparticles (25 wt% with particle size of 100–200 nm; Methods and Fig. 1a) dispersed on MoS_2_ flakes as microwave susceptors to create local hot zones and increase the local reaction temperature above the boiling point of hexane (Supplementary Figs. 1 and 2). Compared with other microwave absorbers such as silicon carbide (SiC), carbon black is less dense and disperses uniformly. This leads to rapid phase transformation and the exfoliation of macroscale MoS_2_ powder, as observed in the solutions shown in Fig. 1b. The solutions contain atomically thin 1T(1T′)-phase MoS_2_ nanosheets (Fig. 1c), which shows the typical atomic force microscopy (AFM) image of flakes with ~1-nm thickness and ~160-nm diameter^12^ (Supplementary Fig. 3 shows the statistical analysis of nanosheet dimensions).

We performed annular dark-field (ADF) scanning transmission electron microscopy (STEM) imaging to confirm the presence of metallic phase. Figure 1d,e shows the ADF-STEM images of MoS_2_ nanosheets synthesized by conventional CE and MWCE for comparison. Atomic-resolution *Z*-contrast observed in the ADF mode and fast Fourier transform were used to identify the different 1T, 2H and distorted 1T′ phases in MoS_2_ nanosheets (Fig. 1f–h)^23,24^. ADF-STEM image of CE MoS_2_ (Fig. 1d) shows mixed 1T(1T′) and 2H phases, consistent with previous reports^23^. By contrast, MWCE MoS_2_ (Fig. 1e and Supplementary Fig. 4a) only shows the metallic 1T(1T′) phases. Fast Fourier transform of the entire CE MoS_2_ region shows two sets of hexagonal diffraction patterns, which are offset by a small angle of 6° due to the low-angle grain boundary at the 1T/2H heterointerface. By contrast, the MWCE sample shows two sets of monoclinic diffraction patterns due to the 60° offset between two 1T′ grains (Fig. 1f). The experimental and simulated diffraction patterns viewed along the [001] direction provides further evidence to our phase assignments (Supplementary Fig. 4b). Although the 1T and distorted 1T′ phases are structurally distinct, they are (semi)metallic in nature with much higher electrical conductivity, in stark contrast to the semiconducting 2H phase^17,25^. For the electrochemical applications explored in this work, including hydrogen evolution reaction (HER), supercapacitors and lithium–sulfur (Li–S) batteries, the key material properties are high electrical conductivity and the availability of catalytically active sites. Both 1T and 1T′ phases provide these properties^26^, and thus, they will both be referred to as metallic 1T(1T′) phase in the following sections.

**Fig. 2:**
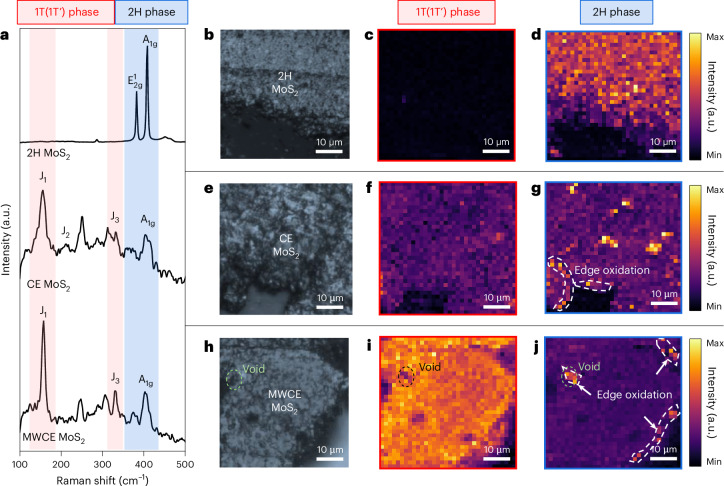
Raman spectroscopy of chemically exfoliated MoS_2_ nanosheets. **a**, Typical Raman spectra of 2H, CE and MWCE MoS_2_ nanosheets. **b**–**d**, Optical image (**b**) and *J*_1_ (**c**) and *A*_1g_ (**d**) peak intensity maps of 2H MoS_2_. **e**–**g**,Optical image (**e**) and *J*_1_ (**f**) and *A*_1g_ (**g**) peak maps of CE MoS_2_. **h**–**j**, Optical image (**h**) and *J*_1_ (**i**) and *A*_1g_ (**j**) peak maps of MWCE MoS_2_. The 2H-phase-like features are only observed at the edges in the MWCE sample, in contrast to the CE sample.

We then performed Raman mapping to assess the phase concentration uniformity in larger samples. Raman spectra of different types of MoS_2_ are shown in Fig. 2a. Precursor 2H-phase samples show the typical in-plane ${E}_{2g}^{1}$ and out-of-plane ${A}_{1g}$ modes of MoS_2_, whereas the chemically exfoliated MoS_2_ nanosheets show *J* peaks that are characteristic of the 1T(1T′) phase. In the predominantly 1T(1T′)-phase samples, the ${E}_{2g}^{1}$ and ${A}_{1g}$ peaks are less pronounced than the *J* peaks^27^. In addition, the CE samples show the *J*_2_ peak that originates from the phase boundaries between the 1T(1T′) and 2H phases^28^. By contrast, the *J*_2_ peak is absent in the MWCE samples and the *J*_1_ and *J*_3_ peaks are sharper and more prominent. The Raman maps of 2H, CE and MWCE MoS_2_ nanosheets are shown in Fig. 2b–j. In the 2H-phase precursor powder samples, only the ${E}_{2g}^{1}$ and ${A}_{1g}$ peaks are observed (Fig. 2c,d). CE MoS_2_ mostly displays the 1T(1T′) phase (Fig. 2f), with spots of the 2H phase observed within the sample and at the edges of the nanosheets in which oxidation and reconstruction are more likely to occur (Fig. 2g). The MWCE samples show a uniform 1T(1T′) phase (Fig. 2i) with minute 2H-phase concentration observed only at the edges of the film and around a void (Fig. 2j).

**Fig. 3:**
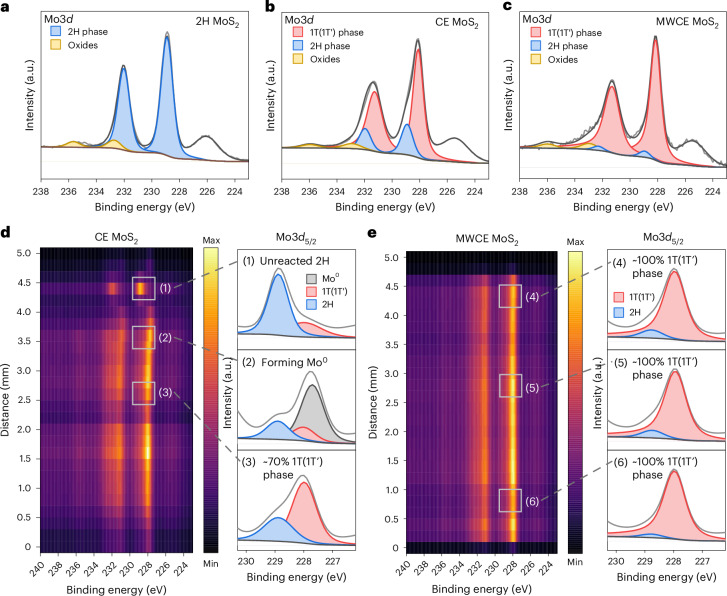
XPS characterization of MoS_2_. **a**–**c**, Mo3*d* XPS spectra of 2H (**a**), CE (**b**) and MWCE (**c**) MoS_2_. **d**, Left: XPS line scan at equidistant points of CE MoS_2_. Right: typical spectra corresponding to unreacted 2H phase, elemental Mo and mixture of 1T(1T′)/2H phases along the line-scan positions labelled as (1), (2) and (3), respectively. **e**, Left: similar line scan to **d**, but for MWCE MoS_2_. No unreacted 2H phase, elemental Mo or mixture of 1T(1T′)/2H phases are observed. Right: typical spectra shown at locations (4), (5) and (6) corresponding to the pure 1T(1T′) phase.

X-ray photoelectron spectroscopy (XPS) was used to quantify the phase concentration and obtain chemical information about the MoS_2_ nanosheets. XPS Mo3*d* peaks are shown in Fig. 3a–c. The MoS_2_ precursor powder shows signals of a pure 2H phase with the usual native oxides. CE MoS_2_ is a mixture of the 1T(1T′) and 2H phase. By contrast, MWCE MoS_2_ consists of the 1T(1T′)-phase Mo3*d*_5/2_ and Mo3*d*_3/2_ peaks (228.0 and 231.1 eV, respectively)^12^. The 2H-phase fraction from native oxides and edges is ~26% in CE MoS_2_ and ~4% in the MWCE samples. Supplementary Fig. 5 shows the Mo L_3_ X-ray absorption spectra for 2H, CE and MWCE MoS_2_. The pre-edge shoulder at 2,522.8 eV representing unoccupied Mo4*d* states in the 1T(1T′) phase is observed for both CE and MWCE MoS_2_.

XPS line scans (Supplementary Fig. 6) of CE and MWCE MoS_2_ films over 5 mm are shown in Fig. 3d,e. For CE MoS_2_, three different typical signals observed at various locations on the sample are labelled as (1), (2) and (3) in Fig. 3d. Location (1) shows the 2H phase from unreacted precursors. The 227.8 eV peak at location (2) is from elemental Mo formed due to the overlithiation of MoS_2_. Location (3) shows the 1T(1T′)-phase peak. The XPS analysis reveals that the CE samples contain ~70% 1T(1T′) phase and ~30% 2H phase. By contrast, the XPS line scan in Fig. 3e of the MWCE samples shows a very uniform 1T(1T′)-phase signal. Signals from positions (4), (5) and (6) are essentially the same and are representative of all the spectra measured along the line. Deconvolution of the Mo3*d*_5/2_ peak for the MWCE samples shows nearly 100% 1T(1T′) phase with no identified decomposition to elemental Mo. Spectra from all points in the line scan are provided in Supplementary Fig. 6a,b.

**Fig. 4:**
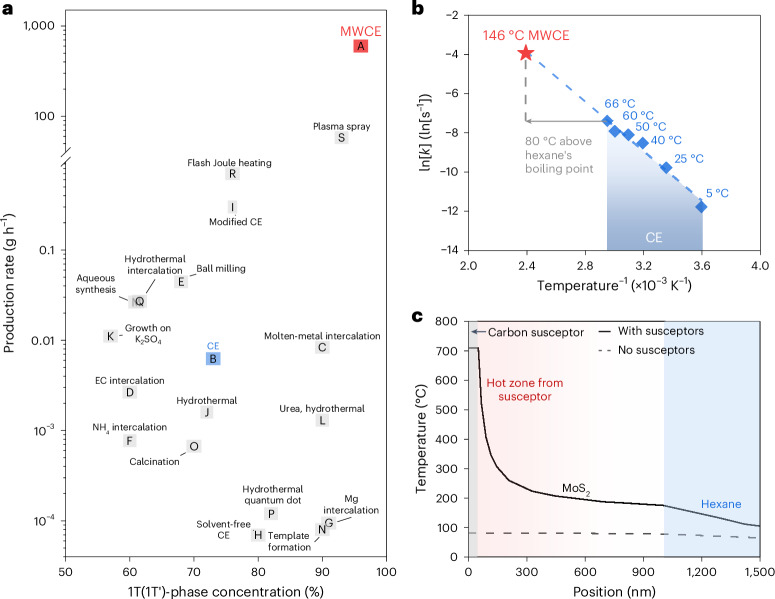
Reaction kinetics and scalability of MWCE. **a**, Comparison of MWCE production rate versus phase concentration for 1T(1T′)-phase MoS_2_ using methods reported in the literature. Letters A–S represent different synthesis methods as labelled next to them (corresponding references are provided in Supplementary Table 1). **b**, Arrhenius plot of the phase conversion rate constant *k* at various temperatures for the CE process. The slope (blue dotted line) yields an activation energy of 0.58 ± 0.06 eV per formula unit of MoS_2_ (56.1 ± 5.8 kJ mol^− 1^); the uncertainty originates from fitting of the slope. Extension of the line to the measured phase conversion rate for MWCE yields a local reaction temperature of 146 °C. **c**, Modelling of the temperature profile of the MoS_2_ flake.

Our results show that MWCE enables the large-scale manufacture of MoS_2_ with a high 1T(1T′)-phase concentration (Fig. 4a). We examine the kinetics of 2H-to-1T(1T′) phase conversion by performing synthesis as a function of temperature (Supplementary Figs. 7–9 show a detailed analysis). With conventional CE, the 1T(1T′)-phase conversion rate reaches a maximum of 0.062% s^−1^ at the boiling point of hexane (66 °C), which is 51 times slower (reaction rate of ~3.2% s^−1^) than for MWCE (>96% 1T(1T′) phase in 30 s). On the basis of this, the activation energy for the 2H-to-1T(1T′) phase transformation was determined to be 0.58 ± 0.06 eV from the Arrhenius plot of the rate constant (*k*) and temperature (Fig. 4b). The plot shows that the reaction in MWCE proceeds at an effective reaction temperature of 146 °C. That is, 80 °C above the boiling point of hexane. Despite this, we do not observe hexane evaporation because the global temperature of the mixture remains well below 66 °C. The short reaction time and large-scale production with MWCE translate to a production rate of 600 g h^−1^, which is several orders of magnitude faster than other synthesis methods (Fig. 4a and Supplementary Table 1). Manufacturing the same amount of material using conventional CE would take over 160 days. Life-cycle analysis also shows substantial energy (~550×) and carbon emission (~120×) savings compared with conventional CE (Supplementary Tables 2–8). Although our microwave is limited to 5-g batches, we do not see any limitations in the manufacture of larger batches with a larger reactor. MWCE also works for other TMDs. The Raman and XPS spectra of MWCE-synthesized 1T(1T′)-phase MoSe_2_ and WS_2_ and the X-ray diffraction (XRD) pattern for MWCE WS_2_ are shown in Supplementary Figs. 10–12.

Finite element simulation (Supplementary Fig. 1) reveals that local heating induced by interfacial polarization is the main contributor to the accelerated reaction kinetics in MWCE (Methods). In heterogeneous systems such as MWCE, the accumulation of charge carriers at material interfaces under the influence of the microwave field creates interfacial dipoles, leading to dielectric loss and localized heating^29^. The simulated electric field strength (Supplementary Fig. 13) demonstrates this effect. The electric field strength around the susceptor interface (13,000 V m^−1^) is about 110% higher than if the susceptor was not present (6,200 V m^−1^). In microwave heating, the heating effect directly relates to the square of the electric field strength (Methods). Thus, in the simulated temperature profile (Fig. 4c), the carbon susceptor reaches a core temperature of 710 °C. This created a localized hot zone of about 1 μm in radius, where the MoS_2_ flake would experience temperatures exceeding 150 °C. By contrast, simulation without a susceptor yields a uniform temperature of 82 °C, consistent with the experimentally determined effective reaction temperature of 76 °C measured in the absence of susceptors (Supplementary Fig. 2). The XRD patterns of MWCE MoS_2_ with carbon susceptors show no remaining 2H crystal structures after 30 s of irradiation (Supplementary Fig. 14), and inductively coupled plasma mass spectroscopy shows nearly full removal of carbon susceptors via centrifugation (Methods and Supplementary Table 9).

**Fig. 5:**
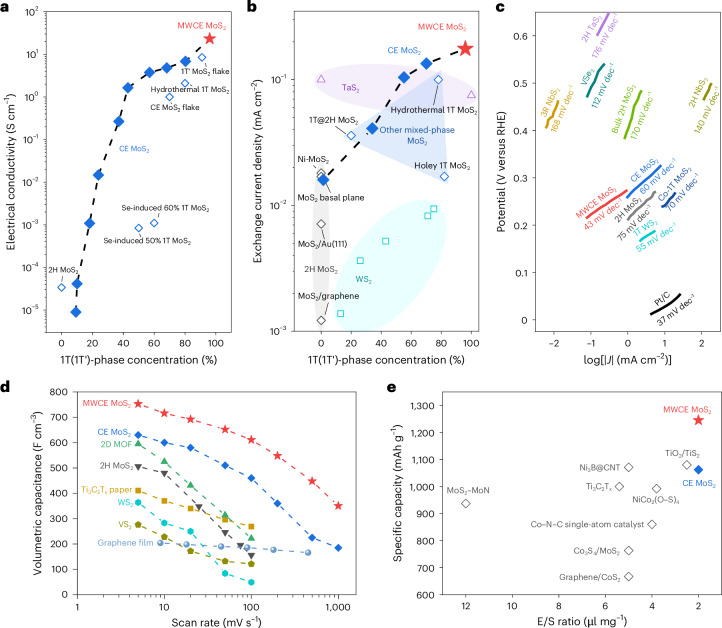
Electrochemical applications of MWCE MoS_2_. **a**, Electrical conductivity as a function of 1T(1T′)-phase concentration for CE and MWCE MoS_2_, and other reported values from the literature. Data include mechanically exfoliated 2H MoS_2_, 50% and 60% 1T MoS_2_ induced by Se insertion^30^, CE MoS_2_ flake^12^, 1T MoS_2_ synthesized via hydrothermal method (hydrothermal 1T MoS_2_) and 1T′ MoS_2_ flake^7^. Data for CE MoS_2_ is taken from our previous study^3^. **b**, Exchange current density as a function of the 1T(1T′)-phase concentration for CE and MWCE MoS_2_, and other TMD-based HER catalysts. Data include 2H MoS_2_ edges on Au(111), graphene, vertically grown Ni-promoted 2H MoS_2_ edges^31^, 2H MoS_2_ basal plane^32^, 1T TaS_2_ (ref. ^33^), 2H TaS_2_ (ref. ^34^), WS_2_ with various 1T-phase concentrations^24^, hydrothermal 1T MoS_2_ (ref. ^35^), holey 1T MoS_2_ (ref. ^36^) and 1T-incorporated 2H MoS_2_ (1T@2H MoS_2_)^37^. **c**, Tafel slopes for CE and MWCE MoS_2_, and other metallic TMD-based catalysts. Data include 2H and 3R NbS_2_ (ref. ^38^), 2H TaS_2_ and vanadium diselenide (VSe_2_)^39^, 1T WS_2_ (ref. ^24^) and Co-doped 1T MoS_2_ (ref.^40^). **d**, Volumetric capacitance of MWCE MoS_2_ and CE MoS_2_ (ref. ^6^), and other two-dimensional-based supercapacitor electrodes (Supplementary Table 11). **e**, Specific capacity in relation to the E/S ratio for practical Li–S pouch-cell cathodes based on CE and MWCE MoS_2_ and other values reported in the literature (Supplementary Table 12).

Electrochemical properties of metallic MoS_2_ are directly correlated with the 1T(1T′)-phase concentration. The MWCE samples show one of the highest electrical conductivities measured (Fig. 5a), which translates into excellent electrochemical performance. To this end, MWCE MoS_2_ exhibits one of the highest exchange current densities for the HER of any TMD catalyst (Fig. 5b). The exchange current density in HER indicates the intrinsic catalytic activity of a catalyst. Figure 5c shows that MWCE MoS_2_ catalysts exhibit a Tafel slope of 43 ± 4 mV dec^−1^, which is close to the lowest values measured for MoS_2_ catalysts (Supplementary Table 10 provides an exhaustive list of Tafel slopes from MoS_2_ catalysts). The low Tafel slopes of MWCE MoS_2_ catalysts suggest fast reaction kinetics due to the high electrical conductivity, and that Heyrovsky electrochemical desorption is the rate-determining step^2,3^. Moreover, MWCE MoS_2_ demonstrates phase stability even after 300 HER cycles (Supplementary Fig. 15).

Supercapacitors with 70% 1T(1T′)-phase CE MoS_2_ nanosheets show a volumetric capacitance^6^ of 650 F cm^−3^ at a scan rate of 5 mV s^−1^. In comparison, supercapacitors based on MWCE MoS_2_ show a higher volumetric capacitance of 753.0 ± 3.6 F cm^−3^. The improved conductivity of the MWCE 1T(1T′) phase also improves the rate capability, as shown by the near-ideal rectangular cyclic voltammetry curves at various scan rates (Supplementary Fig. 16). MWCE MoS_2_ shows a much better rate capability (60% capacitance retention at 500 mV s^−1^) than that of the CE MoS_2_ (35% capacitance retention at 500 mV s^−1^) and various other two-dimensional materials (Fig. 5d and Supplementary Fig. 17).

Recently, we demonstrated that metallic MoS_2_ is an excellent sulfur cathode host for Li–S batteries^5^. In practical pouch-cell batteries, MWCE MoS_2_ cathodes with high areal sulfur loading (7.6 mg cm^−2^) under exceptionally lean electrolyte conditions (electrolyte-to-sulfur (E/S) ratio, 2.0 µl mg^−1^) demonstrate a specific capacity of 1,245 ± 16 mAh g^−1^ (Supplementary Fig. 18a). This is higher than that of CE MoS_2_ cathodes under the same working conditions (1,070 mAh g^−1^), together with a considerably higher cycling stability (Supplementary Fig. 18b). Figure 5e shows that compared with other Li–S cathodes in practical pouch-cell batteries, MWCE MoS_2_ demonstrates one of the highest specific capacities and maintains the lowest electrolyte volume.

## Conclusion

In summary, we have developed the MWCE process for the rapid and scalable production of pure-phase metallic MoS_2_ nanosheets. Kinetic analyses of the phase transformation reveal that the extremely fast reaction is enabled by the localized reaction temperature above the solvent boiling point. By using this method, a production rate of 600 g h^−1^ for metallic MoS_2_ nanosheets can be achieved. As-synthesized metallic MoS_2_ nanosheets demonstrate excellent performance as electrodes for HER, supercapacitors and Li–S batteries. Our research opens opportunities for the scalable synthesis of high-quality metallic two-dimensional TMD materials.

## Methods

### Chemical exfoliation of MoS_2_

Chemical exfoliation of MoS_2_ was done similar to our previous reports. Bulk 2H MoS_2_ powder (0.3 g; Alfa Aesar) was immersed in hexane (15 ml; Sigma-Aldrich) and *n*-BuLi solution (1.6 M in hexane, 3 ml; Sigma-Aldrich). The mixture was refluxed for 48 h under argon in an 80 °C oil bath. The Li_*x*_MoS_2_ product was then washed in hexane (3 × 50 ml) and exfoliated into nanosheets in deionized water or tetrahydrofuran at a concentration of 2 mg ml^−1^ via ultrasonication. The unreacted 2H precursor was removed via centrifugation after dispersion (1 mg ml^−1^ at 1,500 r.p.m. (280*g*) for 15 min). The cumulative consumed energy was measured with an energy meter over the duration of the CE process. The daily energy usage for CE with a batch size of 0.3 g was 1.6 kWh.

### MWCE of MoS_2_

Bulk 2H MoS_2_ powder (0.3 g) was mixed and ground with porous carbon black powder (Nanografi EC-600JD, 0.1 g) before immersing in hexane (15 ml) and *n*-BuLi solution (3 ml) in a liquid-tight polytetrafluoroethylene vessel (MILESTONE SK-15 100 ml, high pressure) under argon.

The vessel was irradiated under 2.450-GHz microwave for 30 s. The maximum microwave power was set to be 800 W. A magnetic stirrer in the vessel was set to 240 r.p.m. during irradiation. The temperature of the total mixture was controlled to not exceed 65 °C via an infrared temperature sensor (MILESTONE easyTEMP) from the bottom of the vessel. After cooling, the Li_*x*_MoS_2_ product was washed in hexane and exfoliated in deionized water or tetrahydrofuran like that of chemically exfoliated MoS_2_. Carbon black dispersion stability is poor in water and tetrahydrofuran, and can be removed from the MoS_2_ nanosheets together with the unreacted bulk via high-speed centrifugation after dispersion at a concentration of 1 mg ml^−1^. Supernatant containing dispersed MWCE MoS_2_ nanosheets was carefully collected without disturbing the settled solids after centrifugation at 3,000 r.p.m. (1,100*g*) for 15 min. Mass yield of the dispersible nanosheets was >80%. The cumulative consumed energy was calculated based on the power profile output by the instrument (Supplementary Fig. 19).

### MWCE of WS_2_

Microwave exfoliation of WS_2_ is similar to that of MoS_2_. The bulk 2H MoS_2_ powder was replaced with 2H WS_2_ powder (0.3 g; Alfa Aesar).

### MWCE of MoSe_2_

Microwave exfoliation of MoSe_2_ is similar to that of MoS_2_. The bulk 2H MoS_2_ powder was replaced with 2H MoSe_2_ powder (0.3 g; Alfa Aesar).

### Materials characterizations

The STEM samples were prepared by drop casting dilute MoS_2_ nanosheet dispersions in deionized water onto lacey carbon grids. Before imaging, the samples were cleaned in distilled acetone and isopropanol, and then stored in an ultrahigh vacuum for drying. Aberration-corrected STEM measurements were conducted on a probe-corrected Thermo Fisher Spectra 300 device operating at 80 kV. Electron beam with a convergence angle of 25.1 mrad, beam current of 20 pA and dwell time of 5 µs were used during the acquisition. Collection semi-angles of 30–112 mrad were used to form the ADF-STEM images. The simulated ADF-STEM images and diffraction patterns were computed using commercial simulation software Dr. Probe^41^, on single-layer MoS_2_ with 2H, 1T and 1T′ phases, using the same parameters as the experimental STEM setup (80 kV, 30–112-mrad collection, beam convergence angle of 25 mrad). A Crispen-Smooth filter^42^ in DigitalMicrograph software was applied to the STEM images to remove low-frequency amorphous hydrocarbon features and emphasize the high-frequency crystalline features for better clarity in structural analyses.

XPS and scanning electron microscopy samples were prepared by depositing MoS_2_ nanosheets on Cu tape under Ar. XPS measurements were taken on a Thermo Fisher Scientific NEXSA G2 using an Al Kα source with 50-µm probe size. The flood gun was turned ON, and an auto-height program of 300 μm was conducted with a step size of 30 μm. C1*s* was calibrated to 284.6 eV. Scanning electron microscopy measurements were taken on a FEI Nova NanoSEM with a 5-kV beam-accelerating voltage. Raman samples were prepared by depositing MoS_2_ nanosheets on SiO_2_ wafers. Raman spectroscopy was taken on a Renishaw InVia using a 514-nm laser beam. The laser power was set to be 0.1% (<0.5 mW). The grating was selected to be 600 mm^−1^. AFM samples were prepared by drop casting dispersed MoS_2_ nanosheets onto a clean SiO_2_ substrate. AFM was performed on a Bruker Icon system with a SCANASYST-AIR tip under the peak-force mode. A 5 μm × 5 μm scan area was acquired at a 256 pixel × 256 pixel resolution. X-ray absorption spectroscopy measurements were done at the I09 beamline at Diamond Light Source located at Dicot, UK. Exfoliated nanosheets were dispersed and then dropped onto a Cu foil in an inert atmosphere before transferring into the analysis chamber. The incident angle was set to –70°. The XRD measurements were done on a Bruker D8 Advance powder X-ray diffractometer using Cu Kα radiation, equipped with automatic divergence slits and a LynxEye-XE position sensitive detector. Solutions for inductively coupled plasma mass spectroscopy measurements were prepared by completely dissolving MWCE nanosheets with concentrated nitric acid and dilution with type I ultrapure deionized water to a concentration of 45 ng ml^−1^. For electrical measurements, MWCE MoS_2_ thin films were deposited after vacuum filtration on a silicon wafer with a 300-nm oxide top layer. The thickness of the films was measured using AFM. Gold electrodes with 20-μm separation were thermally evaporated, and the conductivity was measured at room temperature in a Lake Shore vacuum probe station using a Keithley 4200-SCS system.

### Model framework

COMSOL Multiphysics simulation couples two physics interfaces: the ‘Electromagnetic Waves, Frequency Domain’ (emw) interface, which solves the time-harmonic Maxwell’s equations for the electric field distribution, and the ‘Heat Transfer in Solids and Fluids’ (ht) interface, which solves the stationary heat transfer equation. The coupling between these physics interfaces is handled by the ‘Electromagnetic Heating’ Multiphysics node, which introduces the volumetric heat generated by microwave absorption as a source term in the heat transfer equation. The volumetric heat source, *Qe* (W m^−^^3^), is given by the equation

$${Qe}=\frac{1}{2}\omega {\varepsilon }_{0}\varepsilon {{\prime\prime} \left|E\right|}^{2},$$

where ω is the angular frequency of microwaves (2.45 GHz), ${\varepsilon }_{0}$ is the permittivity of free space, *ε*″ denotes the imaginary part of the material’s relative permittivity (the dielectric loss factor) and |*E*| is the magnitude of the local electric field strength. The model geometry was constructed to represent a single composite particle within the solvent. It consists of three concentric domains: a central hemispherical domain (*d* = 100 nm) representing the nanosized carbon dot, which acts as the primary microwave susceptor (a single susceptor is used to save computational resources). A sheet domain (2 μm × 2 μm × 0.5 μm) represents the MoS_2_ particle. A larger (*d* = 5 µm), outer spherical domain represents the bulk hexane solvent. A physics-controlled mesh was generated using tetrahedral elements. The mesh was refined in the regions of the composite particle and at the particle–solvent interfaces, where the steepest electric field and thermal gradients were expected. The model was solved using a fully coupled, frequency-domain stationary study to find the steady-state electric field and temperature distributions. An incident electric field amplitude (*E*_o_) of 5,200 V m^−1^ was estimated based on the power and dimensions of the microwave oven used.

### Electrochemical characterizations

HER measurements were conducted in a three-electrode system with 0.5 M of argon-saturated sulfuric acid (H_2_SO_4_). 1T(1T′) MoS_2_ dispersed in deionized water was centrifuged at 18,000 r.p.m. (40,000*g*) to settle and remove Li ions and then freeze dried. The 1T(1T′) MoS_2_ nanosheets were supported on glassy carbon working electrodes, similar to our previous reports^24^. Five sets of working CE MoS_2_ electrodes were annealed in a vacuum at 100 °C for various time durations to achieve different 1T-phase concentrations. The working electrodes were allowed to cool in a vacuum and then tested immediately using an Ag/AgCl reference electrode and a graphite-rod counter electrode. Linear sweep voltammetry was conducted with a scan rate of 5 mV s^−1^. All potentials were normalized to a reversible hydrogen electrode (RHE) using the equation

$${E}_{\mathrm{RHE}}={E}_{\mathrm{Ag}/\mathrm{AgCl}}+{E}_{\mathrm{ref}}+0.059\,{\rm{V}}\times \mathrm{pH},$$

where *E*_RHE_ is the potential relative to RHE, *E*_Ag/AgCl_ is the measured potential relative to the Ag/AgCl electrode and *E*_ref_ is taken to be 0.205 V at room temperature. The exchange current density is extracted from the Tafel slopes of various MoS_2_ materials similar to our previous reports^24^.

Supercapacitor electrochemical measurements were conducted in a symmetric two-electrode electrochemical cell with 1.0 M of H_2_SO_4_ electrolyte. MWCE 1T(1T′) MoS_2_ was dispersed in deionized water and centrifuged at 18,000 r.p.m. (40,000*g*) to settle and remove Li ions and then freeze dried. Thereafter, 1 mg of freeze-dried MWCE 1T(1T′) MoS_2_ was mixed with 0.1 mg of polytetrafluoroethylene in ethanol and loaded onto carbon paper with a diameter of 15 mm. Cyclic voltammetry data were collected between 0 V and 0.80 V with various scan rates from 5 to 1,000 mV s^−1^.

### Li–S battery fabrication

CE or MWCE MoS_2_ nanosheets and precipitated sulfur (Thermo Fisher Scientific) were mixed and annealed under Ar at 155 °C for 12 h at a 1:2.5 mass ratio to obtain the MoS_2_/S composite (71.4 wt% sulfur). The composite was then mixed with a poly(vinylidene fluoride) (MTI Corporation) binder at a 95:5 mass ratio and made into a slurry with *N*-methyl-2-pyrrolidone. The slurry is coated on aluminium (Al) sheets. The areal sulfur loading is controlled to be 7.6 mg cm^−2^. A thinner coating with a controlled areal loading of 1.75 mg cm^−2^ is prepared for Li–S coin cells.

Then, 6 cm × 4.5 cm pouch cells were assembled in a dry room (relative humidity, <0.1%). The MoS_2_ cathode, a Celgard polypropylene separator and a lithium foil anode (100-μm thickness) were stacked and packed into Al-laminated films (MTI Corporation). Al and nickel (Ni) tabs (MTI Corporation) were used for the outward connection of the cathode and anode, respectively. The pouch cell was then transferred into an argon-filled glovebox for the injection of electrolyte and encapsulation. The E/S ratio was controlled to be 2.0 μl mg^−1^. The pouch cells were cycled in the voltage range of 2.8–1.7 V at a rate of 0.1C for stability (1C = 1,672 mAh g^−1^). In coin cells, a Li foil was used as the anode and the E/S ratio is controlled to be 30 µl mg^−1^. The coin cells were cycled in the voltage range of 2.8–1.7 V at a rate of 0.1–1C for rate capability, and the cyclic voltammetry data were collected in the same voltage window with various scan rates from 0.1 to 0.5 mV s^−1^.

## Online content

Any methods, additional references, Nature Portfolio reporting summaries, source data, extended data, supplementary information, acknowledgements, peer review information; details of author contributions and competing interests; and statements of data and code availability are available at 10.1038/s41563-026-02480-2.

## Supplementary information

Supplementary Information Supplementary Figs. 1–19 and Tables 1–12.

## Data Availability

The data that support the findings of this study are available within this Article and its Supplementary Information.
