# Supplementary material for: Scalable manufacture of nearly pure-phase metallic MoS2 nanosheets
Source: Nat Mater. 2026 Jan 29;25(8):1329–35. doi: 10.1038/s41563-026-02480-2 (PMC13421342; doi:10.1038/s41563-026-02480-2)
Supplement: Supplementary file 1 — Supplementary Figs. 1–19 and Tables 1–12. [file 41563_2026_2480_MOESM1_ESM.pdf]

nature materials

Supplementary information https://doi.org/10.1038/s41563-026-02480-2

Scalable manufacture of nearly pure-phase metallic MoS_2 nanosheets

In the format provided by the authors and unedited

Table of Contents

Supplementary Figs. 1-19

Supplementary Tables 1-12

Supplementary Section: Detailed analysis of the reaction temperatures and rates with susceptor concentration

Supplementary Section: Comparative Life Cycle Analysis of Conventional CE and MWCE

Supplementary References

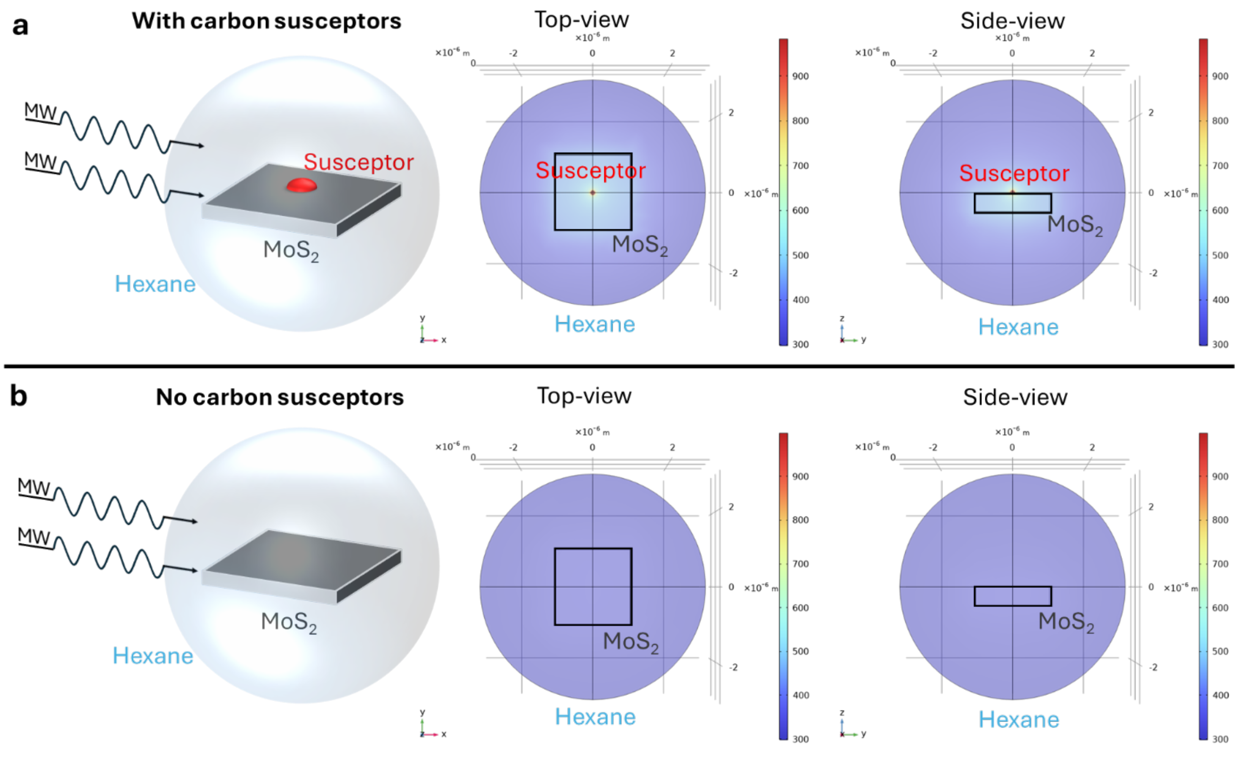

Supplementary Fig. 1: Visualization of simulated temperature profiles of a MoS_2 flake under microwave heating **(a)** with carbon susceptors and **(b)** without susceptors. The peak temperature of the carbon susceptor can reach up to 710 °C. The temperature of MoS_2 is above 150 °C with the susceptor, and 82 °C without the susceptor.

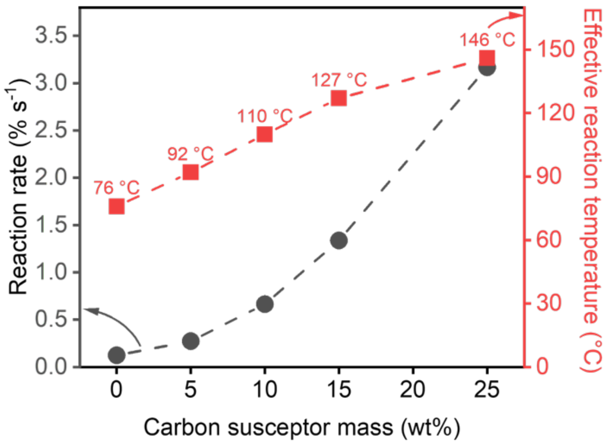

Supplementary Fig. 2: Effect of carbon susceptor concentration on reaction rate and effective reaction temperature. It can be observed that the effective reaction temperature increases almost linearly with the carbon amount until the weight percent reaches 15%. An additional 10% is needed to increase the temperature by 19 °C to the optimal 146 °C.

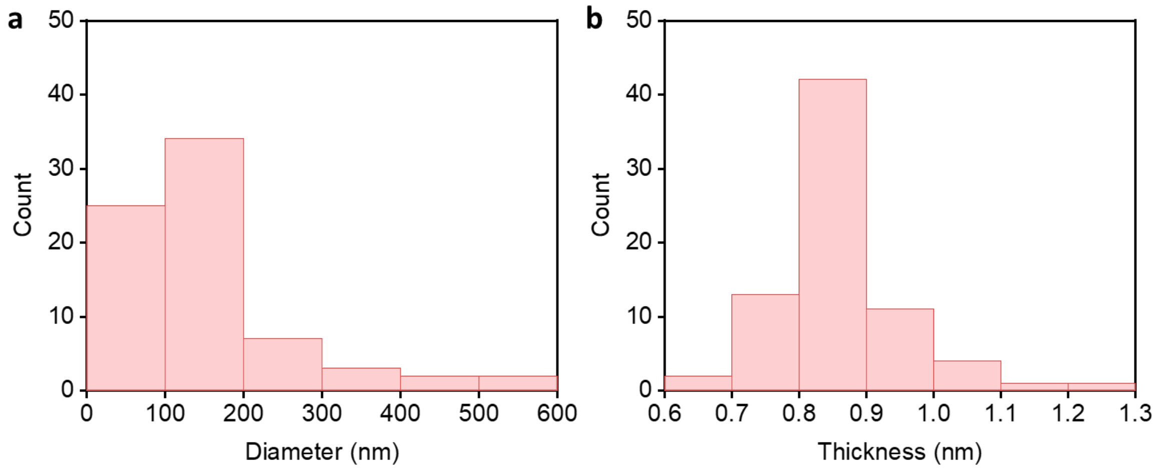

Supplementary Fig. 3: Statistical analysis of MWCE MoS_2 nanosheet dimensions. **(a)** distribution of MWCE MoS_2 nanosheet diameter. **(b)** distribution of MWCE MoS_2 nanosheet thickness.

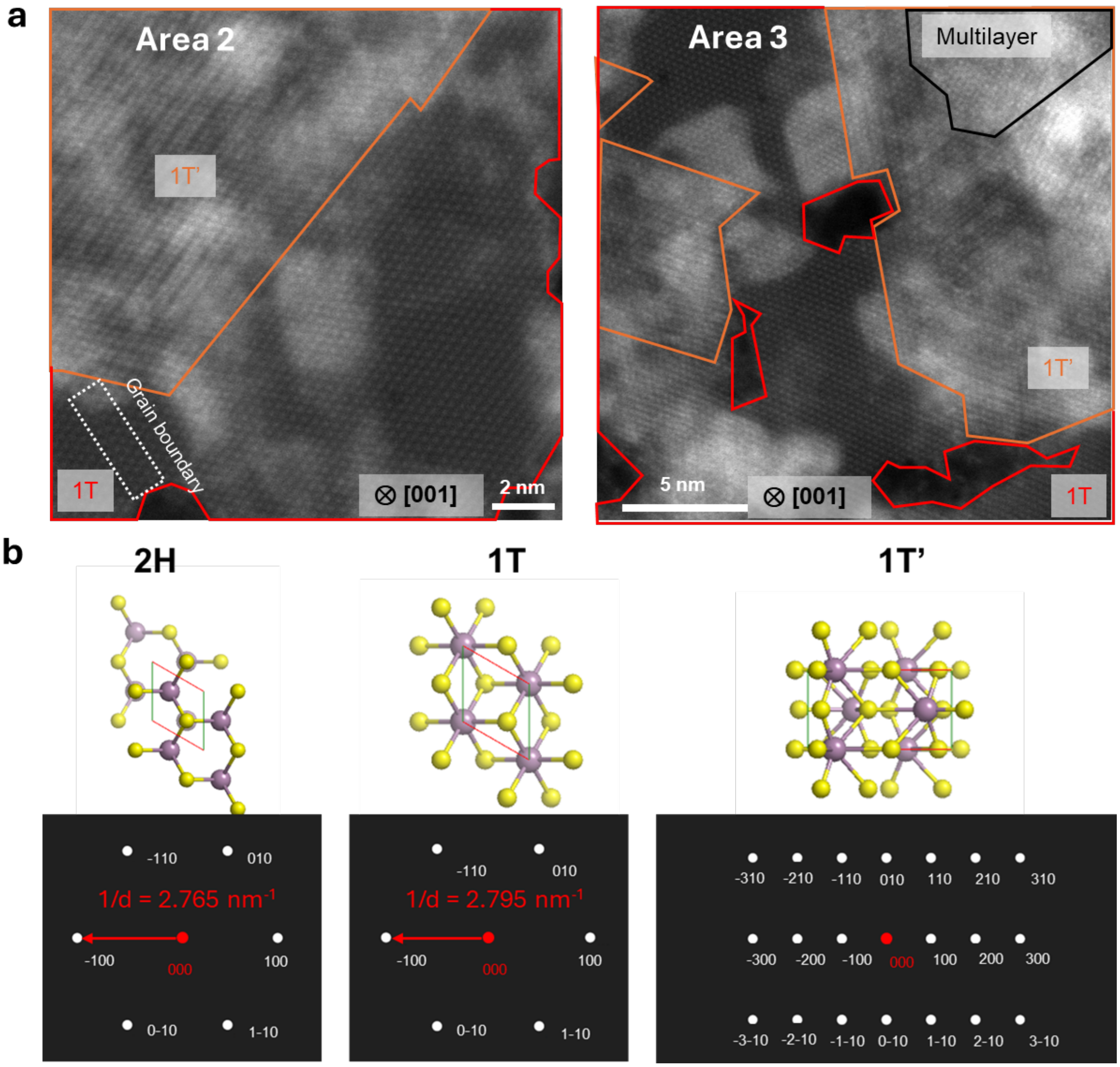

Supplementary Fig. 4: (a) Additional large area ADF-STEM images of MWCE MoS_2 samples showing predominantly a mixture of 1T' (outlined in orange) and 1T phases (outlined in red). Images are captured in the (001) direction. **(b)** Simulated fast Fourier transform of the atomic models of 2H, 1T and 1T' in their respective orientations shown above.

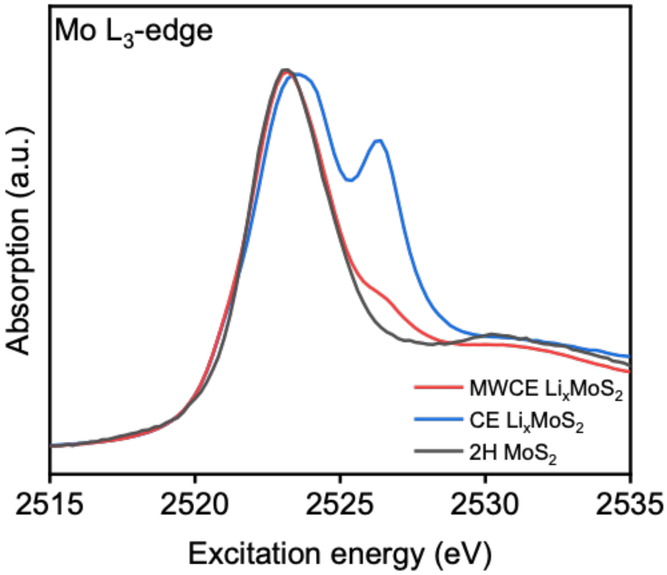

Supplementary Fig. 5: Mo L_3 edge XAS spectra for 2H, CE, and MWCE MoS_2. A strong feature at 2526.4 eV is also observed in the CE MoS_2 sample. This is identified as the oxidation product lithium molybdate (Li_2MoO_4)[1] formed from the increased possibility of exposure to oxygen and moisture impurities in the argon atmosphere over the long reaction time.[2]

\

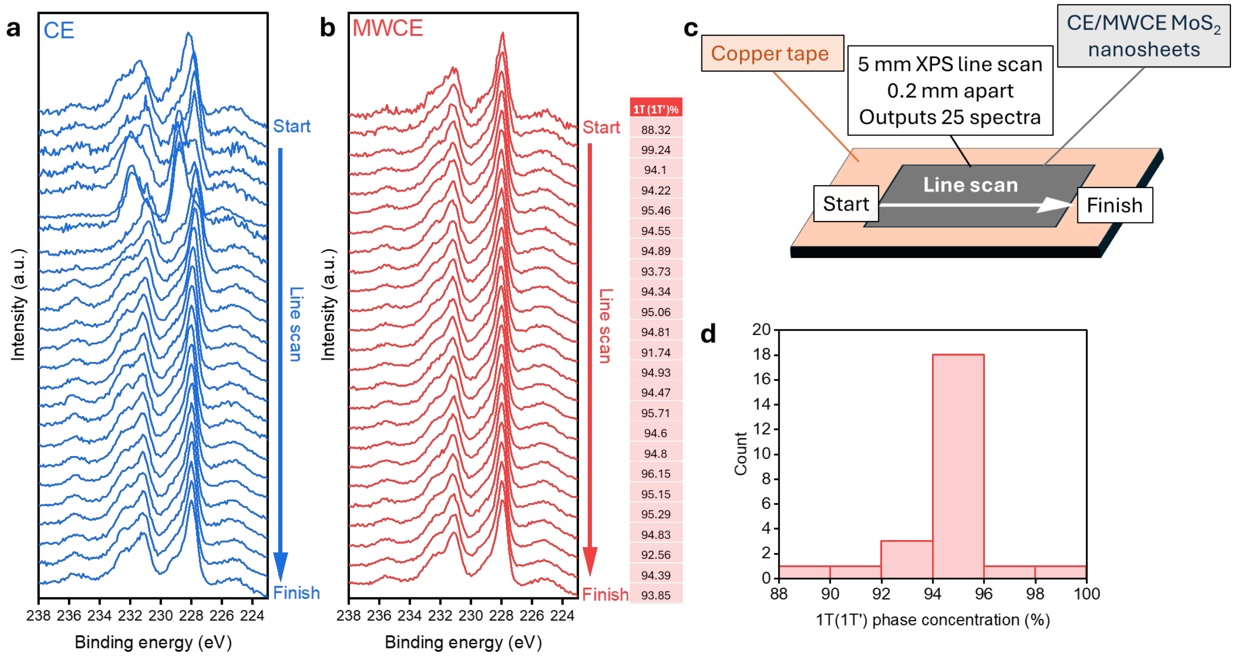

Supplementary Fig. 6: XPS characterization of exfoliated MoS_2 nanosheets. **(a)** Stacked Mo 3*d* spectra for CE MoS_2 at equidistant points along the line scan. The region dominated by the 2H phase can be observed. A slight shift to lower binding energies can also be seen, suggesting a lower oxidation state of the Mo. **(b)** Stacked Mo 3*d* spectra for MWCE MoS_2 at equidistant points along the line scan. **(c)** Schematic for a 5 mm XPS line scan for MoS_2 nanosheets. **(d)** 1T(1T') phase concentration distribution based on the XPS line scan for MWCE MoS_2.

Detailed analysis of the reaction temperatures and rates with susceptor concentration

Determination of order of reaction for CE

A CE reaction at 66 °C was used to determine the order of reaction. Phase concentration of CE MoS_2 at various reaction times were determined by XPS peak deconvolution and fitted to a polynomial function so that the derivative represents the reaction rate at a specific time (Supplementary Fig. 7a). In the excess of n-BuLi, the reaction rate for the 2H to 1T phase transformation can be described in the general form of

$$\frac{d[2H]}{dt} = \frac{-d[1T]}{dt} = k[2H]^n \qquad (1)$$

where $\frac{d[2H]}{dt}$ is the rate of change in 2H phase concentration, $\frac{d[1T]}{dt}$ is the rate of change in 1T phase concentration, k is the temperature-dependent reaction constant, [2H] is the 2H phase concentration, and n is the reaction order. First order reaction kinetics are established when $n \approx 1$, which means the reaction rate is linearly related to the reactant concentration. A power function fitting for the reaction rate with respect to the 2H phase concentration shows n = 1.13 (Supplementary Fig. 7b), which means that CE demonstrates close to first order reaction kinetics, and its reaction rate constant k is does not depend on reactant concentration. Thus, it has the unit min^{-1}.

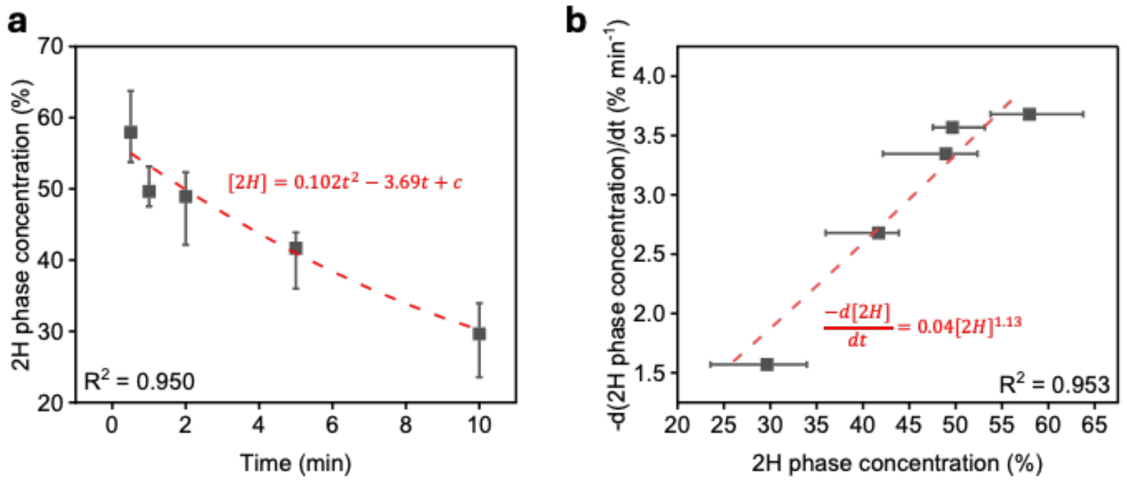

Supplementary Fig. 7: Determination of the reaction order for chemical exfoliation via n-BuLi. Data are presented as mean values ± standard deviation (SD). Statistics were derived from n = 3 independent synthesis experiments. **(a)** Surface 2H phase concentration as a function of reaction time before surface reaction reaches saturation fitted to a polynomial function of time t and constant c with a $R^2 > 0.95$. **(b)** Rate of reaction as a function of 2H phase concentration [2H] fitted as an exponential function with $R^2 > 0.95$. The reaction order is determined to be 1.13, which indicates first-order-reaction behavior for chemical exfoliation via n-BuLi.

Determination of effective reaction temperature and activation energy

Chemical exfoliations of 30 min durations were conducted at temperatures between 5 - 66 °C. Excess n-BuLi at a molar ratio of 2.5 : 1 was added to the reactor via a syringe after MoS_2 and hexane mixture have reached the set temperature. After 30 min, the reactor was quenched in ice, and the CE MoS_2 was washed with hexane immediately. 1T phase concentrations of the MoS_2 products were quantified via peak deconvolution of the Mo 3*d* XPS spectrum (Supplementary Fig. 8). Thus, the rate of reaction can be determined from the reaction time and 1T phase concentration. Respective reaction constants are calculated for each temperature based on the temperature dependent reaction rate and n = 1.13. The relationship between reaction rate constant and temperature follows Arrhenius equation activation energy can be determined. Extension of the Arrhenius plot slope to the reaction rate of MWCE yields the equivalent reaction temperature.

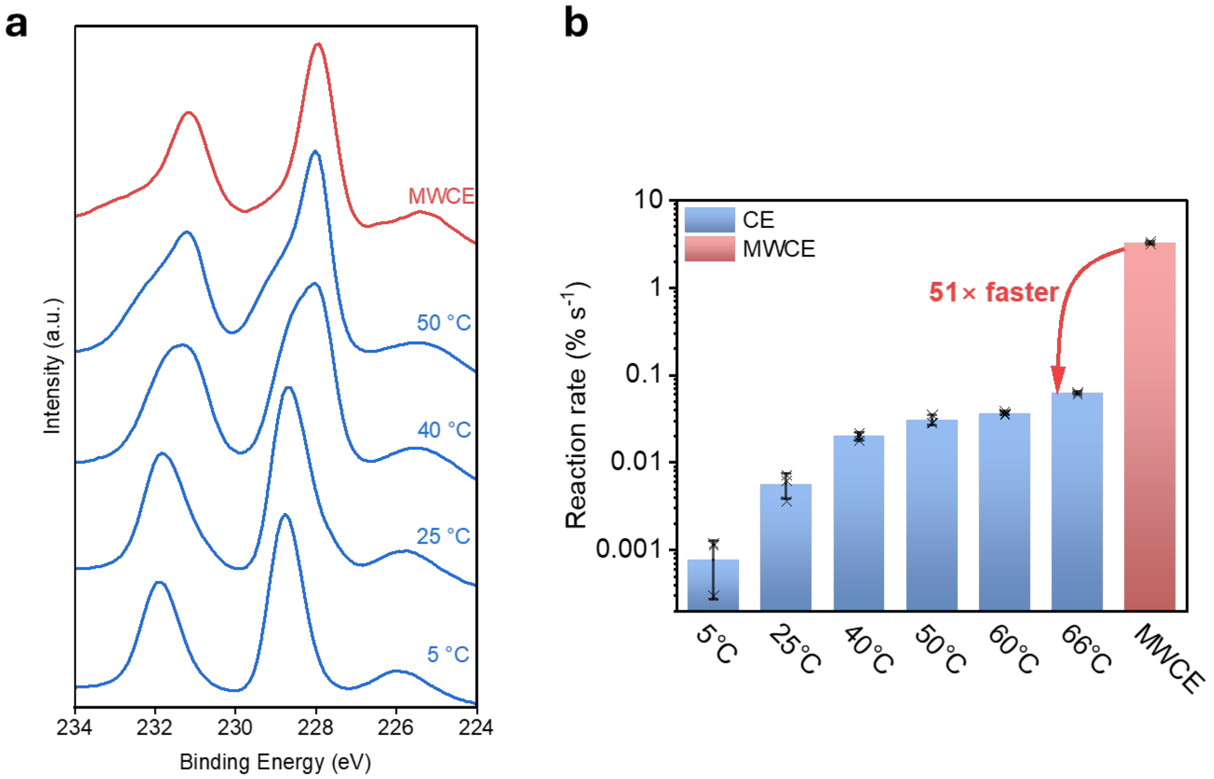

Supplementary Fig. 8: (a) High-resolution Mo 3*d* XPS spectra for MWCE MoS_2 and CE MoS_2 synthesized at various temperatures. The bulk solution temperature for MWCE is 34 °C, however, the strong 1T phase peak suggests full reaction extent and high effective rates compared to conventional CE. **(b)** Reaction rate in the unit of percent-transformed per second (% s^{-1}) for CE MoS_2 synthesized at various temperatures compared to the reaction rate in MWCE. Individual data

points are overlaid on the bars as ×. Data are presented as mean values ± SD. Sample size n = 3 independent synthesis batches for each temperature condition.

Supplementary Fig. 2 shows that without any susceptors, MWCE proceeds at an effective temperature of 76 °C, and every additional 5 weight percent of carbon susceptor increases the effective reaction temperature by about 17 °C until the susceptor reaches 15 weight percent, which corresponds to an effective reaction temperature of 127 °C, and a reaction rate of 1.2 % s^{-1}, about 3 times slower than the optimized 146 °C. Further increase of the susceptor to 50 weight percent results in layers of large agglomerates on the MoS_2 surface, which acts as microwave shields that diminishes heating to the MoS_2 reactant and vaporizes the hexane solution. (Supplementary Fig. 9)

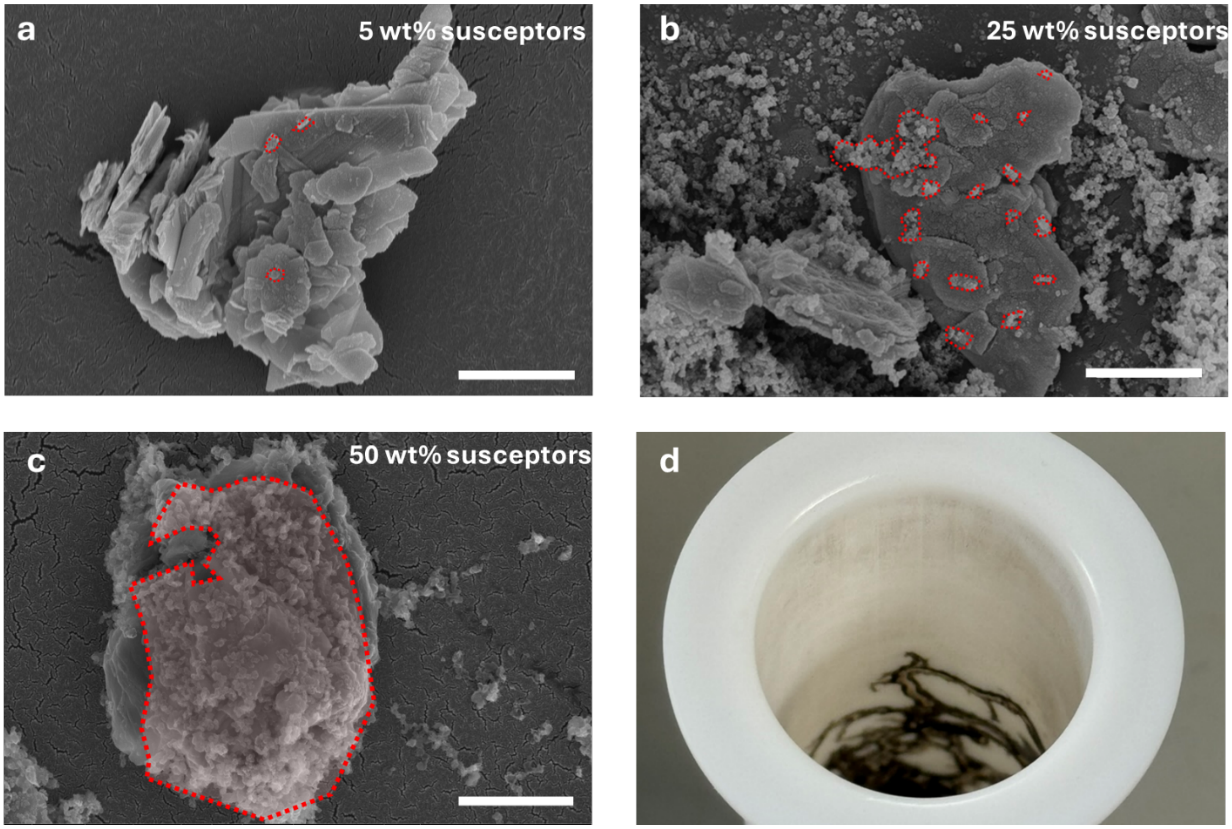

Supplementary Fig. 9: Scanning electron microscopy images of MWCE MoS_2 with **(a)** 5% **(b)** 25%, and **(c)** 50% carbon susceptor. Scale bar, 1 μm. The susceptors of 100 – 200 nm in diameter have been highlighted with dotted red lines. At 50%, the carbon susceptor agglomerates on the MoS_2 surface. **(d)** Reactor damage from vaporized hexane bubbles when susceptor mass percent exceeds 25%. Upon heating, the bulk solution temperature rises quickly, and high-temperature hexane bubbles damage the PTFE reactor.

Supplementary Table 1: Batch size, reaction time, and hourly flow rate of MoS_2 nanosheet synthesis

	Method	1T phase %	Production rate (g h^{-1})	Ref
A	MWCE	96	600	This work
B	CE	73	0.00625	[3]
C	Molten metal intercalation	90	0.00833	[4]
D	Electrochemical intercalation	60	0.00267	[5]
E	Ball Milling	68	0.044	[6]
F	NH_4 intercalation	60	7.72×10^{-4}	[7]
G	Mg intercalation	91	9.44×10^{-5}	[8]
H	Solvent free CE	80	6.94×10^{-5}	[9]
I	Modified CE	76	0.3	[10]
J	Hydrothermal reaction	72	0.0016	[11]
K	Growth on K_2SO_4	57	0.0111	[12]
L	Urea hydrothermal	90	0.0013	[13]
M	Aqeous synthesis	61	0.0265	[14]
N	Template formation	90	8.0×10^{-5}	[15]
O	Calcination	70	6.7×10^{-4}	[16]
P	Hydrothermal quantum dots	82	1.2×10^{-4}	[17]
Q	Hydrothermal intercalation	61.5	0.027	[18]
R	Flash joule heating	76	0.96	[19]
S	Plasma spray	94	58	[20]

Comparative Life Cycle Analysis of Conventional CE and MWCE

This case study presents a comparative life cycle analysis (LCA) for producing metallic 1T phase molybdenum disulfide [1T(1T') MoS_2] from conventional chemical exfoliation using n-butyllithium (n-BuLi), and microwave chemical exfoliation (MWCE). The analysis focuses on three key environmental and economic indicators: total energy consumption, associated carbon dioxide equivalent (CO_2e) emissions, and waste management. The assessment is conducted within the context of a UK laboratory setting, utilizing UK-specific data. Transport costs and capital equipment depreciation are excluded. The functional unit for this comparative LCA is defined as 1 gram of synthesized and post-processed 1T(1T') phase MoS_2 product. All energy consumption, carbon emissions, and waste generations are normalized to this unit, allowing for a direct comparison between methodologies.

The results from this case study are summarized in Supplementary Table 2.

Supplementary Table 2. Summary of case study

Synthesis Method	Total Energy (MJ/g 1T MoS_2)	Carbon Footprint (kg CO_2e/g 1T MoS_2)
Chemical Exfoliation (n-BuLi)	19.52	3.417
Microwave Chemical Exfoliation	0.035	0.0278

Chemical Exfoliation with n-BuLi

The process for this method is adapted from Eda et al. (*Nano Lett.* 2011, 11, 12, 5111–5116), and contains the following steps:

1. Mixing: 0.3 g of bulk MoS_2 powder, 3 mL of 1.6M n-BuLi in hexane solution, and an additional 15 mL of hexane are combined in a round bottom flask.
2. Inert Atmosphere Purging: The flask is purged with Argon (Ar) gas to remove air and moisture. For calculation purposes, a purge for 5 minutes at a flow rate of 1 L/min is assumed (total 5 L Ar).
3. Reaction: The flask is partially submerged in an oil bath maintained at 80 °C, ensuring the reaction solution is kept at the boiling point of hexane (approximately 66 °C). The mixture is stirred continuously for a duration of 48-72 hours; an average of 60 hours is used for this analysis.
4. Condensation: Hexane vapor produced during the reaction is condensed by a water-cooled condenser. Tap water is supplied at a flow rate of 10 mL per minute.
5. Post-Reaction Processing: The product is filtered for 10 minutes using a filtration setup connected to a water pump. After filtration, the product is dried in an oven at 50 °C for 30 minutes.

The energy calculation for this process is shown in Supplementary Table 3, energy-based carbon emission calculation is shown in Supplementary Table 4, and waste-based carbon emission calculation is shown in Supplementary Table 5.

Supplementary Table 3. Energy calculation for Method 1 chemical exfoliation

Process Step	Equipment	Power Rating (kW)	Duration (h)	Duty Cycle (%)	Batch Energy (kWh)
Reaction	Hotplate/ Stirrer		60		0.645
Post-Reaction Filtration	Water Pump	0.05	0.1667 (10 min)	N/A	0.0083
Post-Reaction Drying	Oven	1.6	0.5	20	0.16
**Total Batch Energy**					**0.813**

Energy per gram = 2.92 MJ/0.15 g = 19.52 MJ/g.

Supplementary Table 4. Carbon emission calculation for energy used in chemical exfoliation

Component	Value	Unit
Total Electricity Consumption	0.813335	kWh
Grid Carbon Intensity	0.125	$kgCO_2e/kWh$
**Total Batch CO_2e Emissions**	**0.101667**	**kg CO_2e**

CO_2e emissions per gram = 0.101667 kg CO_2e/0.15 g=0.68 kg CO_2e/g.

Supplementary Table 5. Carbon emission calculation for waste used in chemical exfoliation

Consumable	Mass per g Product (g/g)	Embodied Carbon Factor (kg CO_2e/kg)	Carbon Footprint (kg CO_2e/g Product)
Hexane (Solvent)	77.56	0.62	0.0481
Argon (Purge Gas)	59.46	2.80	0.1665
Tap Water (Cooling)	240,000	0.0105	2.5200
n-BuLi Waste (Treatment via IPA)	0.96	2.45	0.0024
**Total**			**2.737**

Total CO_2e emissions per gram = 0.68 + 2.737 = 3.417 kg CO_2e/g

Microwave Chemical Exfoliation with n-BuLi

The process for this method contains the following steps:

1. Mixing: 0.3 g of bulk MoS_2 powder, 0.1g of ketjen black carbon, 3 mL of 1.6M n-BuLi in hexane solution, and an additional 15 mL of hexane are combined in a microwave vessel in a glovebox. A total batch size of 5 g can be obtained by adding additional vessels.
2. Reaction: The vessels are irradiated under 800W microwave for 30 s.
3. Post-Reaction Processing: The product is filtered for 10 minutes using a filtration setup connected to a water pump. After filtration, the product is dried in an oven at 50 °C for 30 minutes.

The energy calculation for this process is shown in Supplementary Table 6, carbon emission calculation is shown in Supplementary Table 7, and waste calculation is shown in Supplementary Table 8. It should be noted that while microwave chemical exfoliation can achieve ~100% mass yield.

Supplementary Table 6. Energy calculation for Method 5 microwave chemical exfoliation

Process Step	Equipment	Power Rating (kW)	Duration (h)	Duty Cycle (%)	Batch Energy (kWh)
Reaction	Microwave	0.8	0.0083		0.0066
Post-Reaction Filtration	Water Pump	0.05	0.1667 (10 min)	N/A	0.0083
Post-Reaction Drying	Oven	1.6	0.5	20	0.16
**Total Batch Energy**					**0.175**

Energy per gram = 0.175 MJ/5.0 g = 0.035 MJ/g.

Supplementary Table 7. Carbon emission calculation for energy used in MWCE

Component	Value	Unit
Total Electricity Consumption	0.175	kWh
Grid Carbon Intensity	0.125	kgCO_2e/kWh
**Total Batch CO_2e Emissions**	**0.0218**	**kg CO_2e**

CO_2e emissions per gram = 0.0218 kg CO_2e/5.0 g=0.0044 kg CO_2e/g.

Supplementary Table 8. Carbon emission calculation for waste used in MWCE

Consumable	Mass per g Product (g/g)	Embodied Carbon Factor (kg CO_2e/kg)	Carbon Footprint (kg CO_2e/g Product)
Hexane (Solvent)	37.62	0.62	0.0233
n-BuLi Waste (Treatment via IPA)	0.038	2.45	0.0001
**Total**			**0.0234**

Total CO_2e emissions per gram = 0.0044 + 0.0234 = 0.0278 kg CO_2e/g

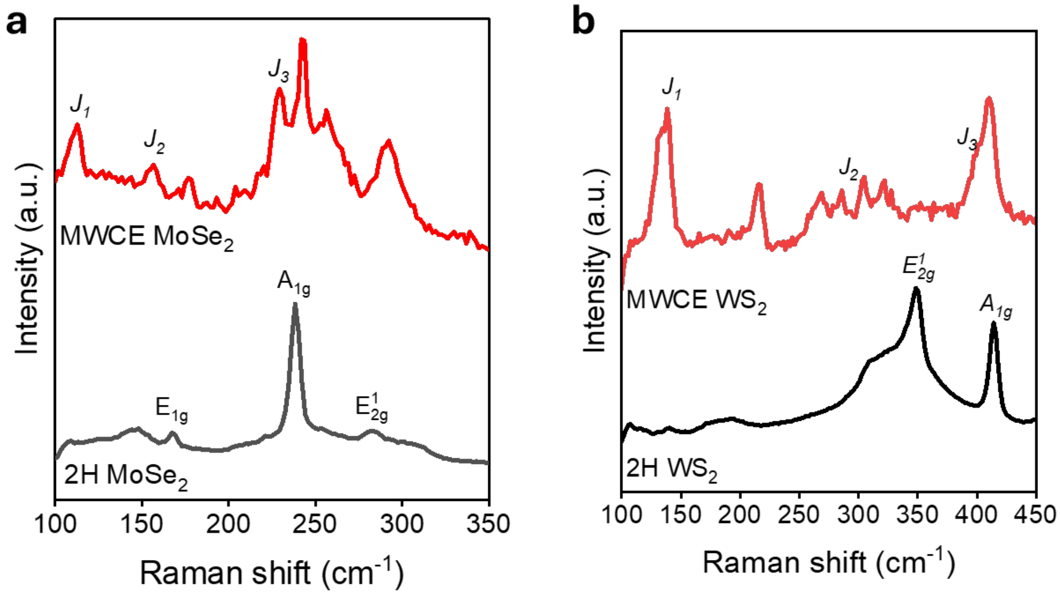

Supplementary Fig. 10: (a) Raman spectra for 2H $MoSe_2$ and MWCE $MoSe_2$, showing the presence of *J*-series peaks characteristic to the 1T phase. **(b)** Raman spectra for 2H WS_2 and MWCE WS_2, showing the presence of *J*-series peaks characteristic to the 1T phase.

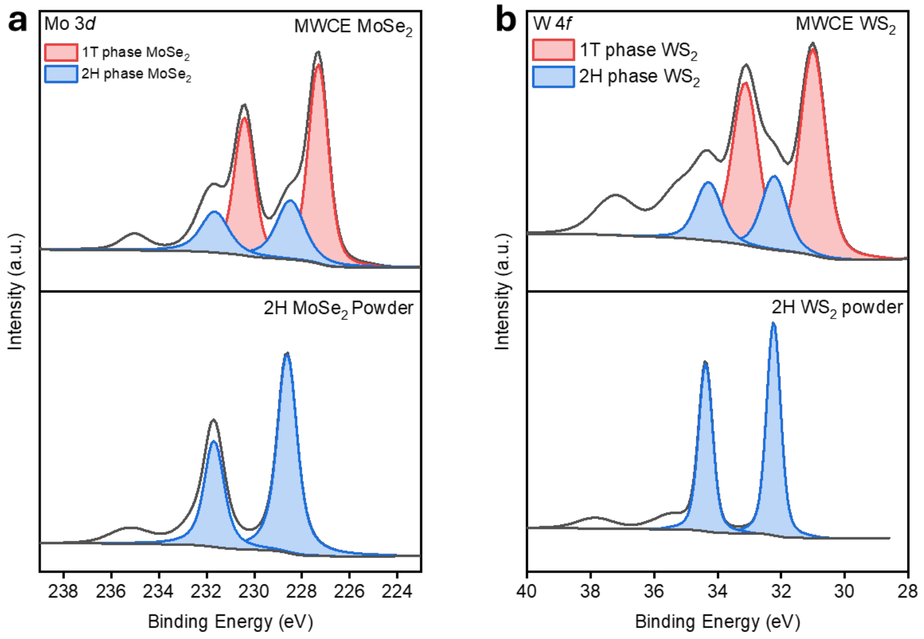

Supplementary Fig. 11: (a) High-resolution Mo 3*d* XPS spectra for MWCE $MoSe_2$ with predominant 1T phase (top) and 2H $MoSe_2$ powder (bottom). **(b)** High-resolution W 4*f* XPS spectra for MWCE WS_2 with predominant 1T phase (top) and 2H WS_2 powder (bottom).

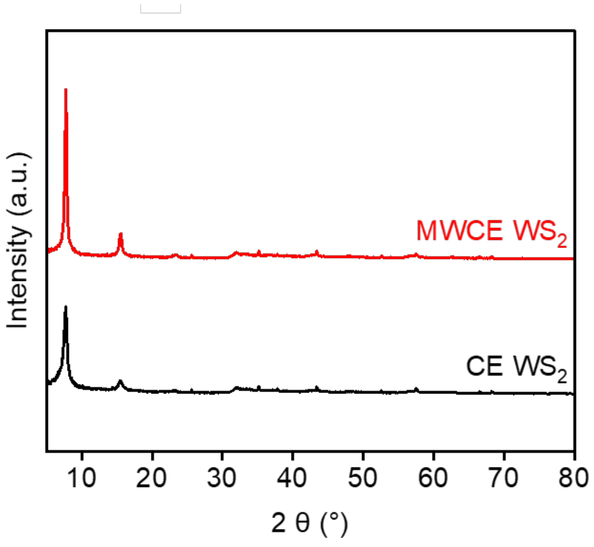

Supplementary Fig. 12: XRD patterns for MWCE and CE WS_2 (with 10 wt% Al_2O_3 reference).

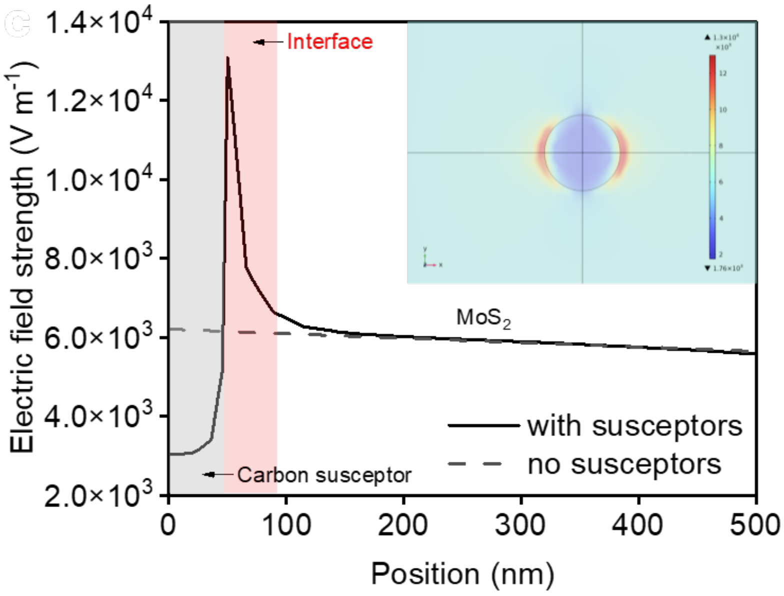

Supplementary Fig. 13: Modelling of the electric field strength at the susceptor/MoS_2 interface.

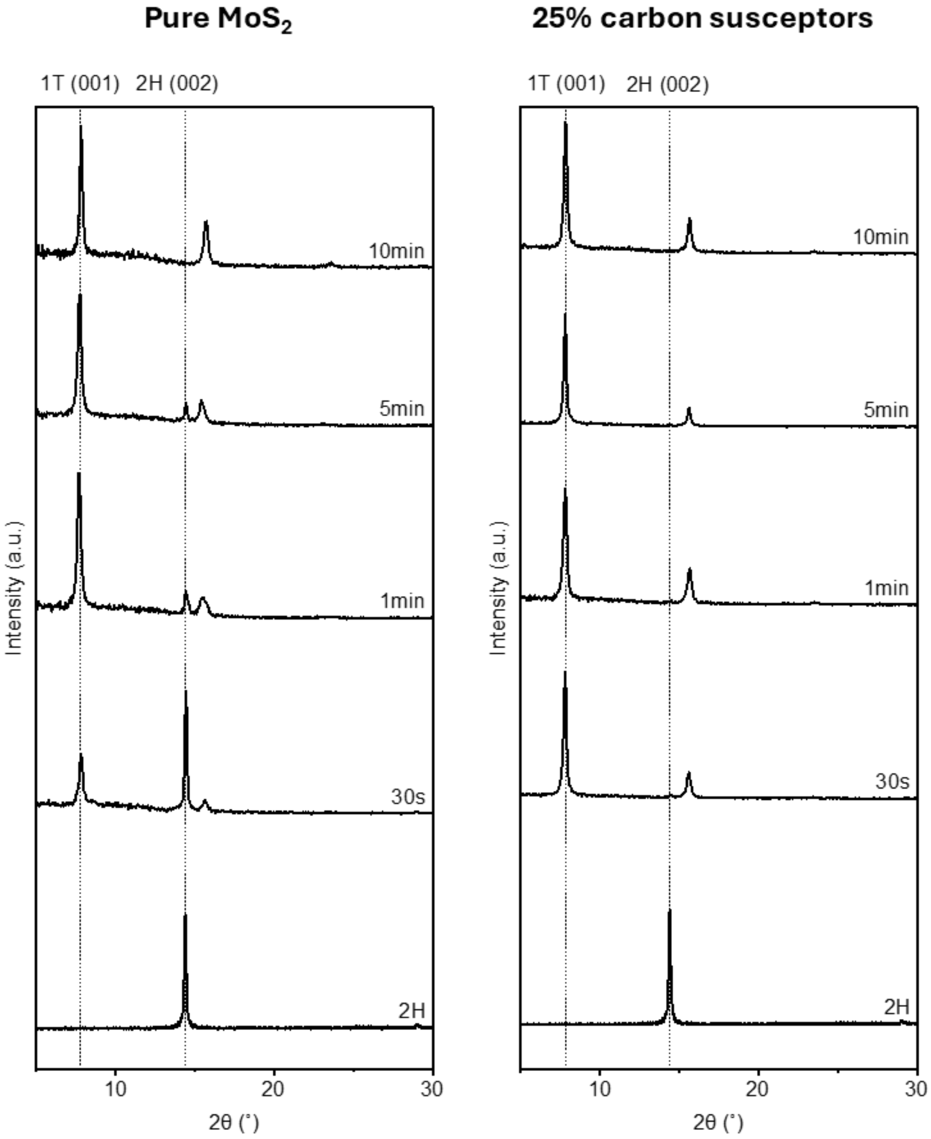

Supplementary Fig. 14: XRD patterns of various reaction times showing the transformation from 2H to 1T(1T') phase for MWCE without (left) and with susceptors (right).

Supplementary Table 9: ICP-MS Mo and C elemental analysis for MWCE MoS_2 with no addition of carbon (reference) and MWCE MoS_2 after carbon removal. They both show negligible amounts of carbon, suggesting nearly full carbon removal after centrifugation.

	Mo concentration (ng mL^{-1})	C concentration (ng mL^{-1})	MoS_2 concentration (ng mL^{-1})	C:MoS_2 mass ratio
**Reference**	44.6 ± 3.30	0.67 ± 0.02	74.3 ± 5.50	0.90%
**MWCE after C removal**	44.0 ± 3.21	0.66 ± 0.02	73.3 ± 5.35	0.90%

Supplementary Table 10: Summary of Tafel slopes of MoS_2 catalysts.

**Catalyst**	**Condition**	**Tafel slope (mV dec^{-1})**	**Highlights**	**Ref**
MWCE MoS_2	acid	43.6 ± 4.0	Large scale manufacture of metallic MoS_2	This work
1T MoS_2	acid	50	Phase engineering	[21]
1T MoS_2	acid	40	Phase engineering	[9]
1T' MoS_2	acid	100	Phase engineering	[22]
1T MoS_2	acid	41	Phase engineering	[23]
1T MoS_2/TiO_{2-x}@Ti	acid	42	Phase engineering	[24]
1T MoS_2@Ti	acid	56	Phase engineering	[24]
Se-1T MoS_2	acid	47	Phase engineering, doping	[25]
1T–2H MoS_2	alkaline	65	Phase engineering, heterostructure	[26]
1T/2H MoS_2	acid	61	Phase engineering	[27]
1T/2H MoS_2	acid	72	Phase engineering	[28]
1T MoS_2 nanosphere	acid	58	Phase engineering	[29]
Pt-1T MoS_2	acid	56	Phase engineering, doping	[30]
1T/2H MoS_2	acid	75	Phase engineering	[31]
1T/2H MoS_2@N-graphene	acid	46	Phase engineering, heterostructure	[31]
V doped-1T MoS_2	acid	54	Phase engineering, doping	[32]
Single S-vacancy MoS_2	acid	48	Surface engineering (defect)	[33]
Single-vacancy MoS_2	acid	44	Surface engineering (defect)	[34]
strained S-vacancy MoS_2	acid	60	Surface engineering (defect)	[35]
Etched MoS_2 basal plane	acid	96	Surface engineering (defect)	[36]
Defect-rich MoS_2 nanosheets	acid	50	Surface engineering (defect)	[37]
defective MoS_2 nanomesh	acid	46	Surface engineering (defect)	[38]

Co doped-MoS_2	alkaline	67	Surface engineering (doping)	[39]
Single-atom Ru doped-MoS_2	acid	21	Surface engineering (doping, vacancy)	[40]
Zn doped-MoS_2	acid	51	Surface engineering (doping)	[41]
Ni doped-MoS_2	acid	81	Surface engineering (doping)	[42]
V doped-MoS_2 nano petals	acid	59	Surface engineering (doping)	[43]
V doped-MoS_2 nano petals	alkaline	89	Surface engineering (doping)	[43]
N doped-MoS_2	acid	41	Surface engineering (doping)	[44]
P doped-MoS_2/N,S doped-rGO	acid	47	Surface engineering (doping), heterostructure	[45]
P doped-MoS_2 nanoflakes	acid	45	Surface engineering (doping)	[46]
MoS_2-WS_2	acid	72	Heterostructure	[47]
MoS_2/WSe_2	acid	76	Heterostructure	[48]
CoS_2-MoS_2	acid	90	Heterostructure	[49]
CoS_2-MoS_2	alkaline	92	Heterostructure	[49]
1T MoS_2-CoS_2	acid	43	Heterostructure, phase engineering	[50]
1T MoS_2-CoS_2	alkaline	60	Heterostructure, phase engineering	[50]
$Cu_{1.94}S$/1T' MoS_2	acid	42	Heterostructure	[51]
NiS_x/MoS_2	alkaline	104	Heterostructure	[52]
$Ni(OH)_2$/MoS_2	alkaline	60	Heterostructure	[53]
1T MoS_2 quantum sheets/$Ni(OH)_2$	alkaline	30	Heterostructure	[54]
MoS_2/NiCo-LDH	alkaline	77	Heterostructure	[55]

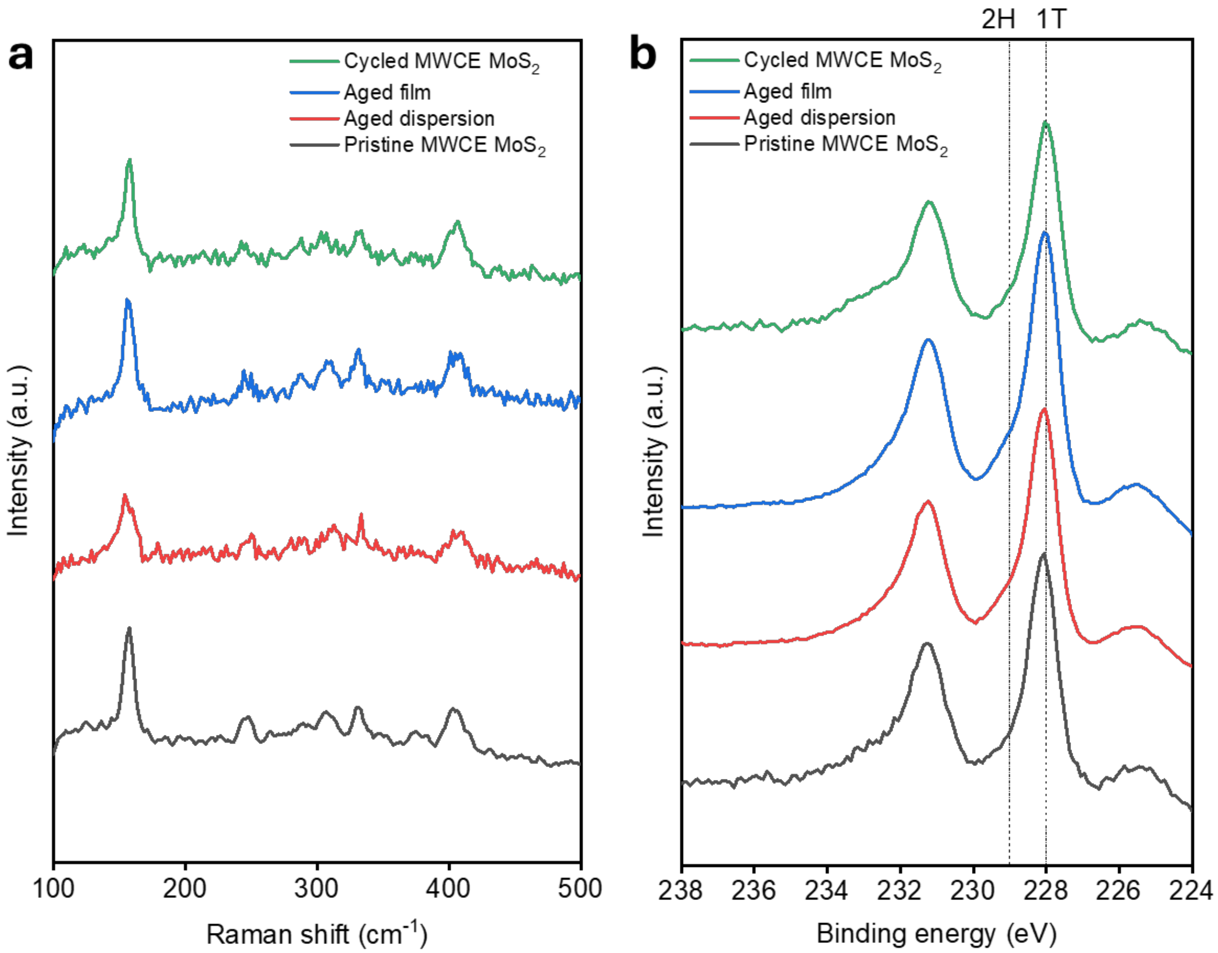

Supplementary Fig. 15: (a) Raman and **(b)** XPS spectra for pristine MWCE MoS_2 nanosheets, film MWCE MoS_2 nanosheets, dispersed MWCE MoS_2 nanosheets which were aged in inert atmosphere for 60 days, and MWCE MoS_2 nanosheet HER electrodes after completing 300 cycles in the span of 12 hours.

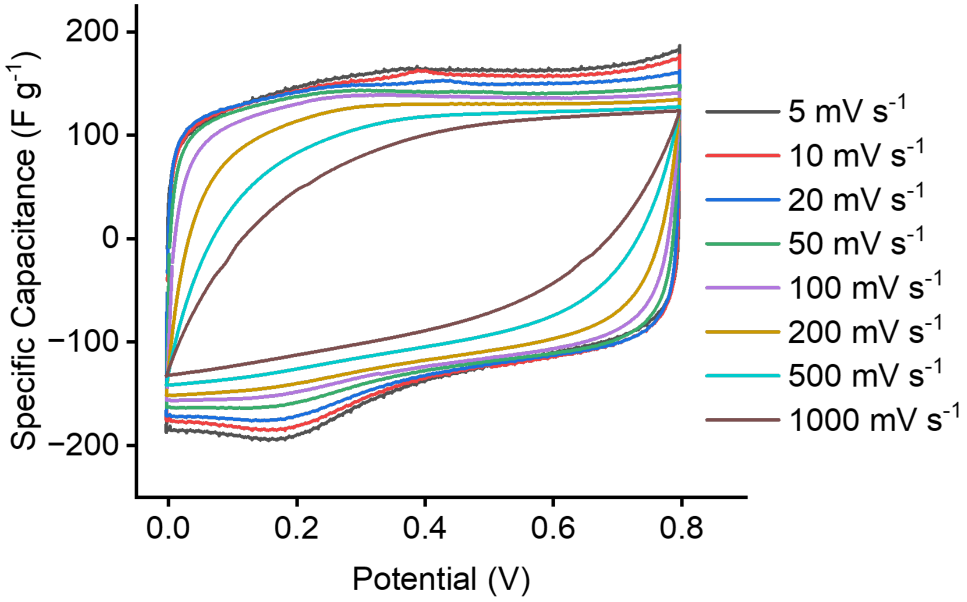

Supplementary Fig. 16: CV of MWCE MoS_2 supercapacitor electrodes in H_2SO_4 from a scan rate of 5 to 1000 mV s^{-1}.

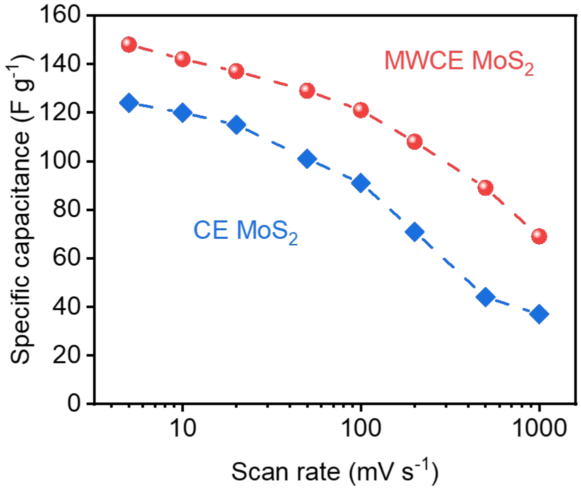

Supplementary Fig. 17: Specific capacitance of MWCE MoS_2 and CE MoS_2.

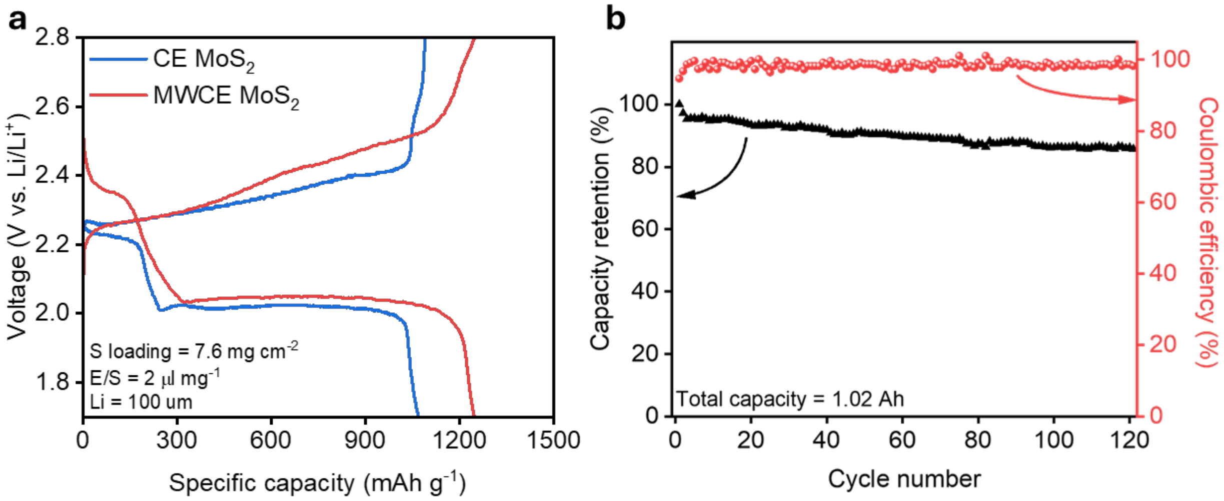

Supplementary Fig. 18: (a) Galvanostatic charge-discharge curves for Li-S pouch cell batteries with cathodes based on CE and MWCE MoS_2 under lean-electrolyte conditions at 0.1 C. **(b)** Coulombic efficiency and capacity retention for a Li-S pouch cell battery with MWCE MoS_2 sulfur host under practical conditions. The Li-S pouch cell battery displayed good cycle retention, losing only 0.12 % capacity per cycle and high coulombic efficiency (> 97%) over the initial 120 cycles.

Supplementary Table 11: Literature data for 2D materials-based supercapacitors

Electrode Material	Reference
MWCE MoS_2	This work
CE MoS_2	[56]
Graphene film	[57]
2H MoS_2 sponge	[58]
2H WS_2	[59]
Vanadium sulfide (VS_2)	[60]
2D Mxene ($Ti_3C_2T_x$)	[61]
2D hexaaminobenzene metal organic framework (HAB-MOF)	[62]

Supplementary Table 12: Areal loading and capacity for practical Li-S batteries

E/S ratio (μL mg^{-1})	Areal Loading (mg S cm^{-2})	Areal Capacity (Ah cm^{-2})	Cathode host material	Reference
2	7.6	9.46	MWCE MoS_2	This work
2	7.6	8.07	CE MoS_2	[63]
5	14.8	11.29	Co_3S_4/MoS_2 nanorod	[64]
2.5	7.5	8.1	TiO_2/TiS_2 heterostructure	[65]
3.8	8.75	8.68	Oxygen-doped $NiCo_2S_4$ [$NiCo_2(O–S)_4$]	[66]
5.4	4.7	4.7	$Ti_3C_2T_x$ MXene	[67]
5	5.6	3.73	graphene/CoS_2 heterostructure	[68]
5	5.6	6.0	Ni_3B/CNT composite	[69]
12	6.4	5.99	MoS_2/MoN heterostructure	[70]
4	2	1.72	Co-N-C single atom catalyst	[71]

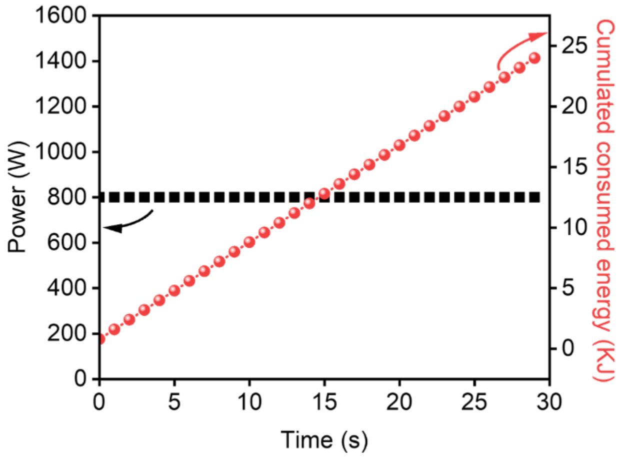

Supplementary Fig. 19: Power profile and cumulated consumed energy for a 30 s MWCE process. The power is controlled to not exceed 800W. Thus, the total cumulated consumed energy is the area under the power curve and determined to be 24 KJ.

References

1 Svyazhin, A. *et al.* Chemical Information in the L(3) X-ray Absorption Spectra of Molybdenum Compounds by High-Energy-Resolution Detection and Density Functional Theory. *Inorg. Chem.* **61**, 869-881 (2022).

2 Yang, Z. J. *et al.* Environmental and Thermal Stability of Chemically Exfoliated LixMoS2 for Lithium–Sulfur Batteries. *Chem. Mater.* **36**, 4829-4837 (2024).

3 Eda, G. *et al.* Photoluminescence from Chemically Exfoliated MoS2. *Nano Lett.* **11**, 5111-5116 (2011).

4 Park, S. *et al.* Phase Engineering of Transition Metal Dichalcogenides with Unprecedentedly High Phase Purity, Stability, and Scalability via Molten-Metal-Assisted Intercalation. *Adv. Mater.* **32**, 2001889 (2020).

5 Ejigu, A., Kinloch, I. A., Prestat, E. & Dryfe, R. A. W. A simple electrochemical route to metallic phase trilayer MoS2: evaluation as electrocatalysts and supercapacitors. *J. Mater. Chem. A* **5**, 11316-11330 (2017).

6 Jian, J. *et al.* Cobalt and Aluminum Co-Optimized 1T Phase MoS2 with Rich Edges for Robust Hydrogen Evolution Activity. *ACS Sustain. Chem. Eng.* **10**, 10203-10210 (2022).

7 Wang, D. *et al.* Swollen Ammoniated MoS2 with 1T/2H Hybrid Phases for High-Rate Electrochemical Energy Storage. *ACS Sustain. Chem. Eng.* **5**, 2509-2515 (2017).

8 Chen, F. *et al.* Unlocking robust lithium storage performance in High 1T-phase purity MoS2 constructed by Mg intercalation. *Nano Energy* **104** (2022).

9 Voiry, D. *et al.* Conducting MoS2 nanosheets as catalysts for hydrogen evolution reaction. *Nano Lett.* **13**, 6222-6227 (2013).

10 Peng, J. *et al.* High phase purity of large-sized 1T′-MoS2 monolayers with 2D superconductivity. *Adv. Mater.* **31**, 1900568 (2019).

11 Liu, Z. *et al.* Vertical nanosheet array of 1T phase MoS2 for efficient and stable hydrogen evolution. *Appl. Catal. B* **246**, 296-302 (2019).

12 Gao, X., Xiong, L., Wu, J., Wan, J. & Huang, L. Scalable and controllable synthesis of 2D high-proportion 1T-phase MoS2. *Nano Res.* **13**, 2933-2938 (2020).

13 Sun, K. *et al.* Targeted bottom-up synthesis of 1T-phase MoS2 arrays with high electrocatalytic hydrogen evolution activity by simultaneous structure and morphology engineering. *Nano Res.* **11**, 4368-4379 (2018).

14 Liu, Q. *et al.* Gram-scale aqueous synthesis of stable few-layered 1T-MoS2: applications for visible-light-driven photocatalytic hydrogen evolution. *Small* **11**, 5556-5564 (2015).

15 Venkateshwaran, S. & Senthil Kumar, S. M. Template-driven phase selective formation of metallic 1T-MoS2 nanoflowers for hydrogen evolution reaction. *ACS Sustain. Chem. Eng.* **7**, 2008-2017 (2018).

16 Sun, D. *et al.* 1T MoS2 nanosheets with extraordinary sodium storage properties via thermal-driven ion intercalation assisted exfoliation of bulky MoS2. *Nano Energy* **61**, 361-369 (2019).

17 Li, X. *et al.* One-step hydrothermal synthesis of high-percentage 1T-phase MoS2 quantum dots for remarkably enhanced visible-light-driven photocatalytic H2 evolution. *Appl. Catal. B* **243**, 76-85 (2019).
18 Wang, D. *et al.* Phase engineering of a multiphasic 1T/2H MoS 2 catalyst for highly efficient hydrogen evolution. *J. Mater. Chem. A* **5**, 2681-2688 (2017).
19 Chen, W. *et al.* Millisecond Conversion of Metastable 2D Materials by Flash Joule Heating. *ACS Nano* **15**, 1282-1290 (2021).
20 Kiran, P. S. *et al.* Scaling up simultaneous exfoliation and 2H to 1T phase transformation of MoS2. *Adv. Func. Mater.* **34**, 2316266 (2024).
21 Yang, J. *et al.* Ultrahigh-current-density niobium disulfide catalysts for hydrogen evolution. *Nat. Mater.* **18**, 1309-1314 (2019).
22 Yu, Y. *et al.* High phase-purity 1T′-MoS2- and 1T′-MoSe2-layered crystals. *Nat. Chem.*.**10**, 638-643 (2018).
23 Geng, X. *et al.* Pure and stable metallic phase molybdenum disulfide nanosheets for hydrogen evolution reaction. *Nature Comm* **7**, 10672 (2016).
24 Zhang, T. *et al.* Phase control and stabilization of 1T-MoS2 via black TiO2− x nanotube arrays supporting for electrocatalytic hydrogen evolution. *J. Energy Chem.* **68**, 71-77 (2022).
25 Jiang, L. *et al.* Se and O co-insertion induce the transition of MoS2 from 2H to 1T phase for designing high-active electrocatalyst of hydrogen evolution reaction. *Chem. Eng. J.* **425**, 130611 (2021).
26 Wang, S. *et al.* Ultrastable in‐plane 1T–2H MoS2 heterostructures for enhanced hydrogen evolution reaction. *Advanced energy materials* **8**, 1801345 (2018).
27 Liu, Z. *et al.* Heterogeneous nanostructure based on 1T-phase MoS2 for enhanced electrocatalytic hydrogen evolution. *ACS Appl. Mater. Interfaces* **9**, 25291-25297 (2017).
28 Zhang, Y., Kuwahara, Y., Mori, K., Louis, C. & Yamashita, H. Hybrid phase 1T/2H-MoS 2 with controllable 1T concentration and its promoted hydrogen evolution reaction. *Nanoscale* **12**, 11908-11915 (2020).
29 Wang, S. *et al.* One-step synthesis of 1T MoS2 hierarchical nanospheres for electrocatalytic hydrogen evolution. *ACS App. Energy Mater.* **5**, 11705-11712 (2022).
30 Li, Y. *et al.* Synergistic Pt doping and phase conversion engineering in two-dimensional MoS2 for efficient hydrogen evolution. *Nano Energy* **84**, 105898 (2021).
31 Zang, X. *et al.* 1T/2H mixed phase MoS2 nanosheets integrated by a 3D nitrogen-doped graphene derivative for enhanced electrocatalytic hydrogen evolution. *ACS App. Mater. Interfaces* **12**, 55884-55893 (2020).
32 Li, M. *et al.* Vanadium doped 1T MoS2 nanosheets for highly efficient electrocatalytic hydrogen evolution in both acidic and alkaline solutions. *Chem. Eng. J.* **409**, 128158 (2021).
33 Wang, X. *et al.* Single-atom vacancy defect to trigger high-efficiency hydrogen evolution of MoS2. *J. Am. Chem. Soc.* **142**, 4298-4308 (2020).
34 Yang, J. *et al.* Single atomic vacancy catalysis. *ACS nano* **13**, 9958-9964 (2019).
35 Li, H. *et al.* Activating and optimizing MoS2 basal planes for hydrogen evolution through the formation of strained sulphur vacancies. *Nat. Mater.* **15**, 48-53 (2016).

36 Wang, Z. *et al.* Controllable etching of MoS2 basal planes for enhanced hydrogen evolution through the formation of active edge sites. *Nano Energy* **49**, 634-643 (2018).
37 Xie, J. *et al.* Defect-rich MoS2 ultrathin nanosheets with additional active edge sites for enhanced electrocatalytic hydrogen evolution. *Adv. Mater*, 5807-5813 (2013).
38 Li, Y. *et al.* Engineering MoS2 nanomesh with holes and lattice defects for highly active hydrogen evolution reaction. *App. Catal. B* **239**, 537-544 (2018).
39 Li, Z. *et al.* Confined synthesis of MoS2 with rich co-doped edges for enhanced hydrogen evolution performance. *J. Energy Chem.* **70**, 18-26 (2022).
40 Zhang, J., Xu, X., Yang, L., Cheng, D. & Cao, D. Single-atom Ru doping induced phase transition of MoS2 and S vacancy for hydrogen evolution reaction. *Small Methods* **3**, 1900653 (2019).
41 Shi, Y. *et al.* Energy level engineering of MoS2 by transition-metal doping for accelerating hydrogen evolution reaction. *J. Am. Chem. Soc.* **139**, 15479-15485 (2017).
42 Zhang, H., Yu, L., Chen, T., Zhou, W. & Lou, X. W. Surface modulation of hierarchical MoS2 nanosheets by Ni single atoms for enhanced electrocatalytic hydrogen evolution. *Adv. Func. Mater.* **28**, 1807086 (2018).
43 Bolar, S. *et al.* Optimization of active surface area of flower like MoS2 using V-doping towards enhanced hydrogen evolution reaction in acidic and basic medium. *App. Catal. B* **254**, 432-442 (2019).
44 Xiao, W. *et al.* Dual-functional N dopants in edges and basal plane of MoS2 nanosheets toward efficient and durable hydrogen evolution. *Adv. Energy Mater.* **7**, 1602086 (2017).
45 Guruprasad, K., Maiyalagan, T. & Shanmugam, S. Phosphorus doped MoS2 nanosheet promoted with nitrogen, sulfur dual doped reduced graphene oxide as an effective electrocatalyst for hydrogen evolution reaction. *ACS App. Energy Mater* **2**, 6184-6194 (2019).
46 Chen, A. *et al.* Phosphorus-doped MoS2 hollow microflakes for enhanced electrocatalytic hydrogen evolution. *Mater. Lett.* **233**, 246-249 (2018).
47 Vikraman, D. *et al.* Improved hydrogen evolution reaction performance using MoS2–WS2 heterostructures by physicochemical process. *ACS Sus. Chem. Eng.* **6**, 8400-8409 (2018).
48 Vikraman, D. *et al.* Fabrication of MoS2/WSe2 heterostructures as electrocatalyst for enhanced hydrogen evolution reaction. *App. Surf. Sci.* **480**, 611-620 (2019).
49 Chen, B. *et al.* Fabrication of CoS2-MoS2 heterostructure via interface engineering toward efficient dual-pH hydrogen evolution. *J. Alloys. Compd.* **948**, 169655 (2023).
50 Feng, Y. *et al.* 3D 1T-MoS2/CoS2 heterostructure via interface engineering for ultrafast hydrogen evolution reaction. *Small* **16**, 2002850 (2020).
51 Yi, L. *et al.* Tailoring Copper Single-Atoms-Stabilized Metastable Transition-Metal-Dichalcogenides for Sustainable Hydrogen Production. *Angew. Chem., Int. Ed.*, e202414701

52 He, Z. *et al.* NiS x@ MoS 2 heterostructure prepared by atomic layer deposition as high-performance hydrogen evolution reaction electrocatalysts in alkaline media. *J. Mater. Res.* **35**, 822-830 (2020).
53 Zhang, B. *et al.* Interface engineering: the Ni (OH) 2/MoS2 heterostructure for highly efficient alkaline hydrogen evolution. *Nano Energy* **37**, 74-80 (2017).
54 Chen, W. *et al.* Achieving rich and active alkaline hydrogen evolution heterostructures via interface engineering on 2D 1T-MoS2 quantum sheets. *Adv.Func. Mater.* **30**, 2000551 (2020).
55 Hu, J. *et al.* Nanohybridization of MoS2 with layered double hydroxides efficiently synergizes the hydrogen evolution in alkaline media. *Joule* **1**, 383-393 (2017).
56 Acerce, M., Voiry, D. & Chhowalla, M. Metallic 1T phase MoS2 nanosheets as supercapacitor electrode materials. *Nat. Nanotechnol.* **10**, 313-318 (2015).
57 Li, Z. *et al.* Tuning the interlayer spacing of graphene laminate films for efficient pore utilization towards compact capacitive energy storage. *Nat. Energy* **5**, 160-168 (2020).
58 Balasingam, S. K., Lee, M., Kim, B. H., Lee, J. S. & Jun, Y. Freeze-dried MoS 2 sponge electrodes for enhanced electrochemical energy storage. *Dalton Trans.* **46**, 2122-2128 (2017).
59 Ratha, S. & Rout, C. S. Supercapacitor electrodes based on layered tungsten disulfide-reduced graphene oxide hybrids synthesized by a facile hydrothermal method. *ACS App. Mater. Interfaces* **5**, 11427-11433 (2013).
60 Pandit, B., Bommineedi, L. K. & Sankapal, B. R. Electrochemical engineering approach of high performance solid-state flexible supercapacitor device based on chemically synthesized VS2 nanoregime structure. *J. Energy Chem.* **31**, 79-88 (2019).
61 Lukatskaya, M. *et al*. Cation Intercalation and High Volumetric Capacitance of Two-Dimensional Titanium Carbide. *Science* **341**,1502-1505 (2013).
62 Feng, D. *et al.* Robust and conductive two-dimensional metal− organic frameworks with exceptionally high volumetric and areal capacitance. *Nat. Energy* **3**, 30-36 (2018).
63 Li, Z. *et al.* Lithiated metallic molybdenum disulfide nanosheets for high-performance lithium–sulfur batteries. *Nat. Energy* **8**, 84-93 (2023).
64 Zhou, W. *et al.* One-dimensional confined p–n junction Co 3 S 4/MoS 2 interface nanorods significantly enhance polysulfide redox kinetics for Li–S batteries. *J. Mater. Chem. A* **11**, 926-936 (2023).
65 Nguyen, V. P. *et al.* Intercalation-Conversion Hybrid Cathode Enabled by MXene-Driven TiO2/TiS2 Heterostructure for High-Energy-Density Li–S Battery. *Small Structures*, 2400196 (2024).
66 Li, Y. *et al.* High-density oxygen doping of conductive metal sulfides for better polysulfide trapping and Li2S-S8 redox kinetics in high areal capacity lithium–sulfur batteries. *Adv. Sci.* **9**, 2200840 (2022).

67 Zhang, T. *et al*. A flexible design strategy to modify Ti3C2Tx MXene surface terminations via nucleophilic substitution for long-life Li-S batteries. *J. Energy Chem*. **74**, 349-358 (2022).
68 Li, H. *et al*. Interface covalent bonding endowing high-sulfur-loading paper cathode with robustness for energy-dense, compact and foldable lithium-sulfur batteries. *Chem. Eng. J.* **412**, 128562 (2021).
69 Xiao, W., Yoo, K., Kim, J. H. & Xu, H. Breaking Barriers to High-Practical Li-S Batteries with Isotropic Binary Sulfiphilic Electrocatalyst: Creating a Virtuous Cycle for Favorable Polysulfides Redox Environments. *Adv. Sci.* **10**, 2303916 (2023).
70 Wang, S. *et al*. Insight into MoS2–MoN heterostructure to accelerate polysulfide conversion toward high-energy-density lithium–sulfur batteries. *Adv. Energy Mater.* **11**, 2003314 (2021).
71 Zhao, C. *et al*. A high-energy and long-cycling lithium–sulfur pouch cell via a macroporous catalytic cathode with double-end binding sites. *Nat. Nanotechnol.* **16**, 166-173 (2021).
